# Supplementary material for: SIZE study: study protocol of a multicentre, randomised controlled trial to compare the effectiveness of an interarcuair decompression versus extended decompression in patients with intermittent neurogenic claudication caused by lumbar spinal stenosis
Source: BMJ Open. 2020 Oct 6;10(10):e036818. doi: 10.1136/bmjopen-2020-036818 (PMC7539610; doi:10.1136/bmjopen-2020-036818)
Supplement: Supplementary data [file bmjopen-2020-036818supp001.pdf]

APPENDIX 1

Informed consent materials/form

Interarcuaire decompressie versus laminectomie bij neurogene claudicatie.

- Ik heb de informatiebrief gelezen. Ook kon ik vragen stellen. Mijn vragen zijn voldoende beantwoord. Ik had genoeg tijd om te beslissen of ik meedoe.
- Ik weet dat meedoen vrijwillig is. Ook weet ik dat ik op ieder moment kan beslissen om toch niet mee te doen of te stoppen met het onderzoek. Daarvoor hoef ik geen reden te geven.
- Ik geef toestemming voor het informeren van mijn huisarts/de specialist die mij behandelt dat ik meedoe aan dit onderzoek.
- Ik geef toestemming voor het opvragen van informatie bij de specialist die mij behandelt over de wervelkanaalstenose.
- Ik geef toestemming voor het verzamelen en gebruiken van mijn gegevens voor de beantwoording van de onderzoeksvraag in dit onderzoek.
- Ik weet dat voor de controle van het onderzoek sommige mensen toegang tot al mijn gegevens kunnen krijgen. Die mensen staan vermeld in deze informatiebrief. Ik geef toestemming voor die inzage door deze personen.

<voor zover van toepassing:>

- Ik geef toestemming voor het informeren van mijn huisarts en/of behandelend specialist van onverwachte bevindingen die van belang (kunnen) zijn voor mijn gezondheid.
- Ik geef □ **wel**

  □ **geen**
  toestemming om mijn persoonsgegevens langer te bewaren en te gebruiken voor toekomstig onderzoek op het gebied van mijn aandoening en de onderzochte behandelwijze
- Ik geef □ **wel**

  □ **geen**
  toestemming om mij na dit onderzoek opnieuw te benaderen voor een vervolgonderzoek.
- Ik wil □ **wel**

  □ **niet**
  geïnformeerd worden over welke behandeling ik heb gehad/in welke groep ik zat.

- Ik wil meedoen aan dit onderzoek.

Naam proefpersoon:

Handtekening: Datum : __ / __ / __

Ik verklaar dat ik deze proefpersoon volledig heb geïnformeerd over het genoemde onderzoek.

Als er tijdens het onderzoek informatie bekend wordt die de toestemming van de proefpersoon zou kunnen beïnvloeden, dan breng ik hem/haar daarvan tijdig op de hoogte.

Naam onderzoeker (of diens vertegenwoordiger):

Handtekening: Datum: __ / __ / __

Aanvullende informatie is gegeven door:

Naam:

Functie:

Handtekening: Datum: __ / __ / __

----

* Doorhalen wat niet van toepassing is.
