# Supplementary material for: SIZE study: study protocol of a multicentre, randomised controlled trial to compare the effectiveness of an interarcuair decompression versus extended decompression in patients with intermittent neurogenic claudication caused by lumbar spinal stenosis
Source: BMJ Open. 2020 Oct 6;10(10):e036818. doi: 10.1136/bmjopen-2020-036818 (PMC7539610; doi:10.1136/bmjopen-2020-036818)
Supplement: Author's manuscript [file bmjopen-2020-036818.draft_revisions.pdf]

BMJ Open is committed to open peer review. As part of this commitment we make the peer review history of every article we publish publicly available.

When an article is published we post the peer reviewers' comments and the authors' responses online. We also post the versions of the paper that were used during peer review. These are the versions that the peer review comments apply to.

The versions of the paper that follow are the versions that were submitted during the peer review process. They are not the versions of record or the final published versions. They should not be cited or distributed as the published version of this manuscript.

BMJ Open is an open access journal and the full, final, typeset and author-corrected version of record of the manuscript is available on our site with no access controls, subscription charges or pay-per-view fees (http://bmjopen.bmj.com).

If you have any questions on BMJ Open's open peer review process please email info.bmjopen@bmj.com

BMJ Open

SIZE study: design of a multi-centre, randomised controlled trial to compare the effectiveness of an interarcuair decompression versus extended decompression in patients with intermittent neurogenic claudication.

Journal:	*BMJ Open*
Manuscript ID	bmjopen-2020-036818
Article Type:	Protocol
Date Submitted by the Author:	09-Jan-2020
Complete List of Authors:	Arjun Sharma, Jamie; Erasmus Medical Center, Neurosurgery<br>Gadjradj, Pravesh; Erasmusmc, Neurosurgery<br>Peul, Wilco; Leiden University Medical Center, Neurosurgery<br>Tulder, Maurits; University of Amsterdam, Health Sciences<br>Moojen, Wouter; Medical Centre Haaglanden, Neurosurgery; Leiden University Medical Center, Neurosurgery<br>Harhangi, Biswadjiet; Erasmus MC, ; Erasmus Medical Center, Neurosurgery
Keywords:	NEUROSURGERY, Spine < ORTHOPAEDIC & TRAUMA SURGERY, Neurosurgery < SURGERY

SCHOLARONE™
Manuscripts

I, the Submitting Author has the right to grant and does grant on behalf of all authors of the Work (as defined in the below author licence), an exclusive licence and/or a non-exclusive licence for contributions from authors who are: i) UK Crown employees; ii) where BMJ has agreed a CC-BY licence shall apply, and/or iii) in accordance with the terms applicable for US Federal Government officers or employees acting as part of their official duties; on a worldwide, perpetual, irrevocable, royalty-free basis to BMJ Publishing Group Ltd ("BMJ") its licensees and where the relevant Journal is co-owned by BMJ to the co-owners of the Journal, to publish the Work in this journal and any other BMJ products and to exploit all rights, as set out in our licence.

The Submitting Author accepts and understands that any supply made under these terms is made by BMJ to the Submitting Author unless you are acting as an employee on behalf of your employer or a postgraduate student of an affiliated institution which is paying any applicable article publishing charge ("APC") for Open Access articles. Where the Submitting Author wishes to make the Work available on an Open Access basis (and intends to pay the relevant APC), the terms of reuse of such Open Access shall be governed by a Creative Commons licence – details of these licences and which Creative Commons licence will apply to this Work are set out in our licence referred to above.

Other than as permitted in any relevant BMJ Author's Self Archiving Policies, I confirm this Work has not been accepted for publication elsewhere, is not being considered for publication elsewhere and does not duplicate material already published. I confirm all authors consent to publication of this Work and authorise the granting of this licence.

For peer review only

SIZE study: design of a multi-centre, randomised controlled trial to compare the effectiveness of an interarcuair decompression versus extended decompression in patients with intermittent neurogenic claudication.

Article type: design article

J.R.J. Arjun Sharma, BSc[1] , P.S. Gadjradj MD[2], W.C. Peul MD, PhD[2], MBA, M.W. van Tulder PhD[3], W.A. Moojen MD, PhD, MSc[2] ,B.S. Harhangi MD, PhD, MSc[1] for the SIZE-study group

1. Department of Neurosurgery, Erasmus MC, University Medical Centre, Rotterdam, The Netherlands
2. Department of Neurosurgery, Haaglanden Medical Centre & Leiden University Medical Centre and Haga Teaching Hospital, The Netherlands

3. Department of Health Sciences, Faculty of Science, Vrije Universiteit Amsterdam, MOVE research institute Amsterdam, Amsterdam, the Netherlands.

For peer review only

Protocol version: 3
Funding sources/sponsor: This work was supported by MRace grant, number 2017-17106 (Institutional Grant of Erasmus Medical Centre)
IRB approval status: MEC- 2018-093
Trial registration/ClinicalTrials.gov: NCT03480893
Conflicts of Interest: None declared.

Abstract word count: 272
Manuscript word count: 3930 [excluding references, figures, and tables]
References: 44
Figures: 0
Tables: 1
Supplementary material: 0
Keywords: Spinal Stenosis/surgery, Decompression, Lumbar Vertebrae/surgery, Humans, Laminectomy
Running title: The SIZE study.

Acknowledgements: the SIZE-study group consists, except for the authors, out of
Drs. H.P. van Putten
Dr. R. van Rolde

#Corresponding author:
B.S. Harhangi MD, PhD, MSc
Department of Neurosurgery
Erasmus Medical Centre, Rotterdam, the Netherlands
Dr. Molewaterplein 40, 3015 GD
Postbus 2040, 3000 CA Rotterdam
b.s.harhangi@erasmusmc.nl

Abbreviations:

LSS: Lumbar Spinal Stenosis

INC: Intermittent Neurogenic Claudication

MRI: Magnetic Resonance Imaging

ODI: Oswestry Disability Index

NRS: Numeric Rating Scale

RMDQ: Roland Morris Disability Questionnaire

TUG: Timed-Up and Go Test

For peer review only

For peer review only - http://bmjopen.bmj.com/site/about/guidelines.xhtml

ABSTRACT

Introduction

Intermittent neurogenic claudication (INC) is often caused by lumbar spinal stenosis (LSS). Laminectomy is considered to be the golden standard of the surgical techniques for LSS. Previous studies have shown that laminectomy can potentially cause lumbar instability. Minimal invasive techniques, preserving midline structures including the bilateral small size interarcuair decompression, are currently available. Due to lack of evidence and consensus, surgeons have to rely on their training and own experiences to choose the best surgical techniques for their patients. Hence an observer and patient blinded multi-centre, randomised controlled trial was designed to determine the effectiveness and cost-effectiveness of bilateral interarcuair decompression versus laminectomy for LSS.

Methods and analysis

174 patients above 40 years with at least 12 weeks of complaints of INC will be recruited. Patients are eligible for inclusion if they have an indication for surgery based on a MRI showing signs of LSS. Patients will be randomised to laminectomy or bilateral interarcuair decompression. The primary outcome is functional status measured with the Roland Morris Disability Questionnaire at 12 months. Secondary outcomes consist of pain intensity, self-perceived recovery, functional status measured with the Oswestry Disability Index, and a physical examination. Outcome measurement moments will be scheduled at 3 and 6 weeks, and at 3, 6, 12, 18, 24, 36 and 48 months after surgery. Physical examination will be performed at 6 weeks, and 12, 24 and 48 months. An economic evaluation will be performed and questionnaires will be used to collect cost data.

Ethics and dissemination

The Medical Ethical Committee of the Erasmus Medical Centre Rotterdam approved this study: NL.65826.078.18. the results will be published in an international peer-reviewed journal.

For peer review only

For peer review only - http://bmjopen.bmj.com/site/about/guidelines.xhtml

ARTICLE SUMMARY

'Strengths and limitations of this study'

- Patient and observer blinded randomised controlled design
- Both patient-reported outcomes and objective functional test outcomes will be measured
- Inclusion of an economic evaluation
- Instability as outcome may not be measurable in two years of follow-up.
- Diversity in terminology on posterior decompression techniques may lead to variability on both surgical techniques.

For peer review only

For peer review only - http://bmjopen.bmj.com/site/about/guidelines.xhtml

INTRODUCTION

Lumbar spinal stenosis (LSS) is described as the narrowing of the diameter of the spinal and nerve root canals, which can cause compression of the neural structures in the canals[1]. LSS is a common medical condition in the aging population and is considered the most common reason for lumbar spine surgery for people over the age of 65[2-4]. Intermittent neurogenic claudication (INC) is the most important symptom of LSS, with complaints of leg pain and heaviness of the legs, usually bilateral, which exacerbate during standing and walking. Apart from leg pain, back pain is often also a symptom[5]. As the symptoms aggravate, the ability to walk can become considerably limited, which is the dominant functional impairment and the most common reason for seeking medical care[6 7].

Conservative treatment, such as physical therapy and pain medication, is the first treatment option and may give some relief of symptoms[8]. When conservative treatment fails, surgical treatment is considered the next option[9 10]. The first technique described to widen the lumbar spinal canal is the laminectomy technique, also known as wide bony decompression , which is a widely used technique and considered the golden standard surgical technique for patients with INC caused by LSS[11]. Given that patients often experience post-operative back pain after laminectomy, it is hypothesized that laminectomy is a ground for potential lumbar instability and iatrogenic scoliosis[12-14]. Performing a procedure with potentially more complications in an elderly population could be considered as doubtful. Hence, less invasive techniques, such as the bilateral small size interarcuair decompression, otherwise known as a bilateral laminotomy, limited bony decompression or interlaminar decompression, were developed and implemented in clinical practice. These techniques require less bone tissue to be removed, which possibly can reduce the prevalence of lumbar instability in these patients and consequently improve functioning[15]. Furthermore, it is believed that, compared to laminectomy, these less invasive techniques have the ability to decompress the spinal stenosis while preserving the spinal integrity. However, the

For peer review only - http://bmjopen.bmj.com/site/about/guidelines.xhtml

risk of an insufficient decompression may be higher, possibly resulting in reoperations due to residual stenosis[15-17].

The presumption is often made that, after a wide decompression, recurrence of the complaints are scarce. The opinions on this matter are diverse and a direct comparison has not yet been performed. Therefore, a randomised controlled trial is needed to clarify whether bilateral interarcuair decompression is effective and cost-effective compared to a classical laminectomy.

Study objectives

The primary objective of this study is to determine whether bilateral interarcuair decompression is more effective than laminectomy in patients with INC caused by LSS. The secondary objective is to evaluate if bilateral interarcuair decompression is more cost-effective than laminectomy.

Study design

The study is designed as a multi-centre, patient and observer blinded randomised controlled trial with an economic evaluation alongside. Participants will be allocated to one of the two groups: laminectomy or interarcuair decompression.

For peer review only

METHODS

Study setting/population

In total 174 patients with a MRI-confirmed LSS will be recruited. All patients who will be included should have an indication for surgery due to LSS with minimally 12 weeks of complaints of INC and failed conservative treatment. Patients will be recruited from two hospitals and one private health clinic in the Netherlands:

- Erasmus Medical Centre in Rotterdam.
- Maasstad Hospital in Rotterdam.
- Park Medical Centre in Rotterdam.

Eligibility criteria

In order to be suitable to participate in this study, patients must meet all of the following criteria:

Inclusion criteria

- At least 12 weeks of complaints of INC, without sufficient response to conservative management.
- Indication for an operation according to consensus of surgeon
- MRI demonstrating LSS
- Age above 40 years
- Sufficient knowledge of the Dutch language in order to comprehend the questionnaires and patient information
- Written informed consent

Exclusion criteria

For peer review only - http://bmjopen.bmj.com/site/about/guidelines.xhtml

Patients who meet any of the following criteria will be excluded from participating in this study.

- History of lumbar spine surgery
- More than 2 lumbar levels needing surgery
- Degenerative spondylolisthesis greater than Meyerding grade I (on a scale of I to V)[18 19]
- Concomitant scoliosis or disc herniation
- Severe comorbid medical disorder (American Society of Anesthesiologists > 3)
- Serious psychopathologic disorder
- Pregnancy
- Active malignancy
- Plans to move abroad during study period.

Eligible patients will be randomised in a 1:1 ratio to the two groups.

Interventions

Intervention: bilateral small size interarcuair decompression

The intervention consists of the interarcuair decompression, otherwise known as a bilateral laminotomy, limited bony decompression or a interlaminar decompression. General anaesthesia is to be administered. A midline skin incision is made, after which the paravertebral muscles are dissected subperiosteally and retracted bilaterally. Decompression will be applied through decompression of the ligamentum flavum and partial laminotomy if necessary. The lateral recess will be opened bilaterally and a partial medial facetectomy will be performed in order to maintain stability of the segments. The lamina will be removed up to the ligamentum flavum. The posterior ligaments are spared.[20] The wound is closed in layers. Patients are operated with a loupe magnification or microscope depending on the surgeon's preference.

For peer review only - http://bmjopen.bmj.com/site/about/guidelines.xhtml

Usual care: conventional laminectomy

Usual care consists of conventional laminectomy, otherwise known as wide bony decompression. General anesthesia is to be administered. A midline skin incision is made over the spinous processes. The laminae of the affected level(s) are exposed subperiosteally, and the supraspinous ligament is incised. The spinous process is removed. The supra and interspinous ligament of the affected level is removed by drill or Kerrison punched. Both laminae are removed at the affected level, leaving the facet joint intact. The lateral recess is opened bilaterally and a medial facetectomy will be performed in order to maintain stability of the segments[15]. To clarify, when a single level stenosis is present (e.g. L4-L5), both laminae L4 and L5 will be removed. When a double level stenosis (e.g. L3-L4 and L4-L5) is present, three laminae (L3, L4 and L5) will be removed. The wound is closed in layers with our without a suction drain. Patients will be operated with loupe magnification or microscope depending on the surgeon’s preference.

Use of co-intervention

Pain medication will be provided to patients after surgery, should this be necessary. Further, the use of co-interventions will be tracked by cost questionnaires, in which medication usage and any health care utilization is monitored throughout the follow-up period.

Outcomes

Primary outcomes

For peer review only - http://bmjopen.bmj.com/site/about/guidelines.xhtml

For peer review only

The primary outcome is a functional status measured using the Roland-Morris Disability Questionnaire (RMDQ). This is a 24-point questionnaire that is designed to assess the functional status in patients with low back pain. This outcome measure has been identified as one of the most commonly used outcomes in a population with chronic low back pain[21-25].

Secondary outcomes

Oswestry Disability Index (ODI)

The ODI is one of the principal condition-specific outcome measures used in de management of spinal disorders[26]. The current version 2.1a will be used[27]. It consists of 10 questions, each with six possible answers and each answer option receives a score of 0 to 5 points, yielding a score range between 0 and 50, which is scaled to a 100% range. The questions focus on a range of daily physical functions and how the back or leg pain is affecting the patient's ability to manage in everyday life.

Numeric Rating Scale for leg and low back pain

This parameter will measure the experienced pain intensity in both legs (affected and non-affected) and back. Pain intensity will be rated on an 11-point scale, varying from 0 representing 'no pain' to 10 'the most terrible pain imaginable'. Patients are not permitted to see the pain score indicated during previous visits. Reliability and validity of NRS have been shown[28].

Short Form 36 (SF-36)

The quality of life will be measured with the Dutch version of the Short Form 36 (SF36). The SF36 has been applied and successfully validated in populations with low back pain[29]. This questionnaire relates to the analysis of the general functional status of the patient. The questionnaire is divided into eight health concepts: (1) physical functioning, (2) role limitations because of physical health problems, (3) bodily pain, (4) social functioning, (5) general mental health (psychological distress and well-being); (6) emotional role limitations; (7) vitality and (8) general health perceptions. The scores of the different

For peer review only - http://bmjopen.bmj.com/site/about/guidelines.xhtml

For peer review only

health concepts are added up into a scale of 0-100. A higher score reflects a better general health condition. The physical and mental health will be assessed separately.

Patients' perceived recovery

Recovery will be measured using a seven-point Likert scale. The score on this scale varies from 'complete recovery' to 'worse than ever'. The outcomes 'complete recovery', 'almost complete recovery' and 'moderate recovery' will be considered as recovered[30].

Patients' satisfaction

Patients' satisfaction of change and satisfaction of treatment will be assessed using a seven-point Likert scale, ranging from 'completely satisfied' to 'completely dissatisfied'. 'Completely satisfied', 'almost completely satisfied' and 'moderately satisfied' will be defined as good outcome.

Physical examination

A physical examination will be performed before surgery, at 6 weeks, 12, 24 and 48 months after surgery. This examination will include a neurologic examination, the Timed Up and Go Test (TUG), the Timed Chair-Stand Test and patient's weight. We will assess if there is a difference in muscle strength between the affected leg and the other leg. If both legs are affected, we will note the muscle strength and monitor the progress.

a. *Neurologic examination*

The neurologic examination will include the patellar and the Achilles tendon reflexes; the muscle strength of the quadriceps, the iliopsoas, the hamstrings, the gastrocnemicus and the tibialis anterior muscle, which will be noted as a difference in strength between both legs. The tendon reflexes have to be measured in a sitting upright position with both feet hanging above the

For peer review only

For peer review only - http://bmjopen.bmj.com/site/about/guidelines.xhtml

floor. We will evaluate if there is a difference in muscle strength between the affected leg and the other leg.

b. Timed Up and Go Test (TUG)

Patients are asked to sit in a chair with their arms resting on the armrests. On direction of the research nurse, patients have to walk as fast as possible (without running) to a wall at 3-m distance of the chair. When they have reached that wall, a turn of 180 degrees is made and they walk directly back to the chair and sit down. The research nurse will measure the time (in seconds) it takes for a patient, to perform this action. The time is stopped when the patients are seated again[31]. Patients need to perform this test, three times. Patients are allowed to wear their regular shoes and use a walking aid, if necessary. The TUG test has been described as a valid, reliable tool in the assessment of objective functional impairment in patients with lumbar degenerative disc disease[32 33]. However, this has not been done for LSS. The validation for LSS will be conducted in this study.

c. Timed Chair-Stand Test

Patients are asked to sit down on an armless chair, firmly placed against a wall, with their arms folded across their chest. They are instructed to place their feet flat on the ground. To become familiarized with the exercise, the patients are asked to stand up and sit down again without using their arms. If assistance is required during this action, or this movement cannot be completed by the patient, the test is abandoned. If the manoeuvre is possible, the patient will be asked to, on the word "go", stand up entirely and sit down again, 5 times as quick as possible. The research nurse will time the 5 movements in seconds from the command "go" to the completed fifth stand. The measured time will be noted as the score. If the patient is unable to perform the test 5 times, a maximum score will be noted of 30 seconds[34 35].

Functional lumbar X-rays

For peer review only - http://bmjopen.bmj.com/site/about/guidelines.xhtml

Functional X-rays will be obtained from all patients. The X-ray will be made from AP and lateral position to assess spondylolisthesis. Degenerative spondylolisthesis is defined as a vertebral slip of at least 3 mm[36]. After the measurement of the vertebral slip, this will be classified according to the Meyerding classification. This will be obtained at 6 weeks postoperatively, for level verification and for verification of correct procedure and after two years postoperatively to look for possible instability.

EuroQol-5-dimension- 5-levels

The EuroQol (EQ-5D-5L) is a validated instrument to assess health-related quality of life (QOL). The tool measures five dimensions:

- Mobility
- Self-care
- Pain/discomfort
- Daily activities
- Anxiety

Each dimension consists of one item, in which five levels are distinguished (no problems, minor problems, moderate problems, major problems, severe problems, unable to). The Dutch tariff of the EQ-5D-5L will be used to calculate the quality-adjusted life years (QALYs)[37 38].

Complication and re-operation incidence

Immediately after surgery, the surgeon will report any perioperative complications such as cerebrospinal fluid leakage, nerve root damage and if the operation was implemented at the wrong vertebral level. Additionally, a systematic assessment of complications (including urinary tract infection, progressive neurological deficit and secondary bleeding) will be carried out by the surgeon and the research nurse shortly after discharge.

For peer review only - http://bmjopen.bmj.com/site/about/guidelines.xhtml

Reoperation is considered a bad outcome. The incidence of reoperation as well as perioperative morbidity will be assessed in both groups, using surgical parameters such as blood loss, time of surgery and length of hospital stay.

We used the SPIRIT checklist when writing our report[39].

Patient and public involvement

No patients involved.

For peer review only

For peer review only - http://bmjopen.bmj.com/site/about/guidelines.xhtml

Table 1: Participant timeline schedule

Table	Visit plan and case report forms											
	Intake	Surgery (t_0)	1-2 day(s) following surgery	FU 3 weeks	FU 6 weeks	FU 3 months	FU 6 months	FU 12 months	FU 18 months	FU 24 months	FU 36 months	FU 48 months
Visit surgeon	x				x							
Research nurse visit	x				x			x		x		x
Randomisation		X										
Surgery		X										
Discharge			x									
Physical examination	x				x			x		x		x
Functional x-rays	x				x					x		
Roland Morris Questionnaire	x			x	x	x	x	x		x	x	x
Oswestry Disability Index (ODI)	x			x	x	x	x	x		x	x	x
NRS leg and back pain	x			x	x	x	x	x		x	x	x
Patient self-received recovery and satisfaction	x			x	x	x	x	x		x	x	x
EuroQol-5D	x			x	x	x	x	x		x	x	x
Cost questionnaires	x			x	x	x	x	x	x	x		
Short Form 36 (SF-36)	x			x	x	x	x	x		x	x	x
Revisit and complications	WITH OCCURRENCE											

For peer review only

Sample Size

The sample size of this study is calculated for the primary endpoint of group difference in the RMDQ at 12 months. According to literature, results obtained after interarcuair decompression should be 3 points lower on the RMDQ compared to those for the laminectomy group in order to detect superiority[40].

Hence the sample size of this study is based on a superiority design, using a delta of 3, an assumed true mean difference of 0 and a pooled standard deviation of 6. Using an alpha of 0.05, and a power of 90%, we calculated a sample size of 69 patients per group. Accounting for a 20% loss in follow-up, we will therefore enrol 174 patients (87 patients per group).

Allocation

Computerized block randomisation will take place after the patient has been anaesthetised. Allocation concealment will be assured by using ALEA, a web-based data system supervised by the Clinical Trial Centre (CTC) of the Erasmus MC. Variable block sizes of 4, 6 and 8 will be used and stratified by treatment centre. Patients will be randomised in a 1:1 ratio to laminectomy or bilateral interarcuair decompression. Each patient will be given a unique study number.

Blinding

All researchers and patients will be blinded for the treatment. This is possible, because there are no fundamental differences between both procedures (e.g. general anaesthesia, spinal incision, inpatient procedure). The statistical analyses will be performed blinded. The surgeon cannot be blinded.

Data collection methods

For peer review only - http://bmjopen.bmj.com/site/about/guidelines.xhtml

All patient reported outcomes will be collected preoperatively and after 3 weeks, 6 weeks, 3 months, 6 months, 12 months, 18 months, 24 months, 36 months and 48 months (see Table 1). Patients will receive an email with a link, reminding them of the upcoming data collection and requesting them to fill in the web-based questionnaires. If the questionnaires are not completed, the patient will receive a reminder after one and two weeks. After two weeks, non-responders will be contacted by phone.

Data management

Data from initial visits to the hospital, follow-up visits and questionnaires will be entered into a database via an electronic data system (Gemstracker). This data will be noted and analysed by using coded information (study numbers) without any personal identifiers. Data will be stored via the secure data management system of the trial coordination centre of the Erasmus MC.

Statistical analysis

Patient characteristics will be summarized per treatment group to determine whether the randomisation was balanced. Continuous variables will be presented using means and standard deviations, or in the case of non-normally distributed variables using medians and interquartile ranges. Normality will be graphically assessed. Categorical variables will be presented using counts and percentages. The primary analysis will study differences in the primary outcome measure (RMDQ). The primary endpoint of interest is 12 months after randomisation. The results of the RMDQ will be assessed using generalized linear mixed models (GLMM), to account for the repeated measurements within patients and the multi-centre stratification.

The secondary outcomes (ODI, NRS leg and back pain score, TUG test, TCST and the SF-36) will be similarly assessed using GLMMs. In all analyses the primary assessment of treatment effect will be the estimate of the main effect within the appropriate model at 12 months, adjusted for the stratification

For peer review only - http://bmjopen.bmj.com/site/about/guidelines.xhtml

factors and main covariates. Time to recovery will be analysed using survival analysis (Cox proportional hazards). At 24, 36 and 48 months, the GLMM repeated-measurement analyses using the compound symmetry covariance structure will be used while *group* (interarcuair and conventional), *time* (moments of measurement) and the *interaction* between Group and Time (Group x Time) will be entered as independent variables. Dependent variables are the primary and secondary outcome parameters. Likert scales for self-reported patient recovery and satisfaction will be assessed using descriptive statistics (counts and percentages) and appropriate plots. Comparisons of Likert scale results at individual time points will be tested using Mann-Whitney U tests. Complications and incidence of re-operations will be assessed using descriptive statistics (counts and percentages). Comparisons between treatment groups for specific complications of interest will be assessed using Chi-squared tests or Fisher's Exact tests. If necessary, missing data will be imputed using multiple imputation techniques.

Economic evaluation

An economic evaluation will be performed from the societal and healthcare perspectives to evaluate the cost-effectiveness of-the bilateral interarcuair decompression compared to a 'classic' laminectomy for patients with INC. When the societal perspective is applied, all costs and consequences relevant to the interventions will be taken into account, whereas only costs accruing to the formal Dutch healthcare sector will be considered when the healthcare perspective is applied. Intervention costs will be estimated using a micro-costing approach. Cost questionnaires will be administered at 3, 6 and 12 weeks covering the entire period and at 6, 12, 18, 24, 36 and 48 months after surgery covering the previous three months. Data will be collected on healthcare utilization, the use of informal care, absenteeism, presenteeism and unpaid productivity losses. Costs will be valued using guideline prices recommended in the Netherlands[41].

For peer review only - http://bmjopen.bmj.com/site/about/guidelines.xhtml

A cost-utility analyses will be conducted with quality adjusted life years (QALYs) as outcome. For estimating QALYs, the patients' EQ-5D-5L health states will be converted into utility scores using the Dutch tariff[38]. Linear interpolation between measurement points will be used to subsequently calculate QALYs. Missing data will be imputed using multiple imputation by chained equations[42]. An incremental cost-utility ratio will be calculated by dividing the difference in costs by the difference in effects. The cost-utility ratio expresses the incremental costs per QALY gained. Bootstrapping techniques will be used to estimate the uncertainty surrounding the cost-effectiveness estimates. Uncertainty will be shown in cost-effectiveness planes and cost-effectiveness acceptability curves, and sensitivity analyses will be performed to test the robustness of the study results.

Data monitoring

This study will be monitored according to a detailed monitoring plan adapted to the risk classification of the Dutch University Federation guidelines. Based on this guideline, the risk classification of this study is regarded negligible. Considerations in this assessment are that this is an investigator-initiated trial, not with vulnerable patients, and while side effects, such as nerve root damage, are known, severe adverse events are extremely rare. Audits may be required by the Medical Ethical Committee or by the regulatory authority inspections and will be granted if necessary. Patients' permission for these audits is obtained with informed consent.

ETHICS AND DISSEMINATION

Research ethics approval

This protocol along with the informed consent forms, recruitment materials and other requested documents will be reviewed and approved by the medical ethical committee of Erasmus MC in Rotterdam with respect to scientific content and compliance with applicable research and human subject regulations.

For peer review only - http://bmjopen.bmj.com/site/about/guidelines.xhtml

Protocol amendments

Any subsequent amendments will be reviewed and approved by the ethical review bodies.

Consent or assent

Surgeons will introduce the trial to patients at the outpatient clinic and will hand them a patient information brochure regarding the main aspects of the trial. Patients will be given five working days to decide if they want to participate in the trial. Trained research nurses will call patients after one week to ask if they wish to partake in the trial. After verbal consent, an appointment will be made with the research nurse, where informed consent forms will be obtained.

Confidentiality

All study-related information will be stored securely at the study site. All participant information will be stored in locked file cabinets with limited access. All reports, data collection (e.g. CRFS) and administrative forms will be entered into an online data system. All these documents will be identified by a coded study number only to maintain the patient confidentiality. These data will be stored for at least 15 years.

Ancillary and post-trial care

Adverse events (AE) and serious adverse events (SAE) will be monitored. SAE will be reported within 24 hours. The sponsor has an insurance which is in accordance with the legal obligations in the Netherlands. The insurance applies to the damage that becomes apparent during the study or within four years after the end of the study.

Dissemination policy

For peer review only

For peer review only - http://bmjopen.bmj.com/site/about/guidelines.xhtml

The final trial results will be communicated to the participants, healthcare professionals, professional organizations and relevant guideline committees in the Netherlands. The results will be published in an international peer-reviewed open-access scientific journal. There are no publication restrictions.

Discussion

In this article, a protocol for a multicentre patient and observer blinded RCT of the SIZE study is presented. Surgical treatment, such as laminectomy or interarcuair decompression, for LSS have been shown to reduce symptoms[9 10]. Due to an abundance of surgical interventions to treat LSS, the surgical management as of now, demonstrates a wide variety of preferred treatments by spine surgeons[43]. To reduce this variety, there is a necessity for randomised controlled trials, to create guidelines on the optimal treatment for LSS. A recent study compared the radiological and clinical results of bilateral interlaminar decompression and laminectomy[44]. However, this study was a single centre study, was not blinded, and did not include a computerized randomisation. Furthermore, there was no data regarding the cost-effectiveness of both surgical interventions. The direct and indirect costs of an intervention are important determinants, that have to be taken into account by the surgeon, during everyday decision-making. In the SIZE study, we intend to include an economic evaluation to estimate the cost-effectiveness and therefore acquire new information on this topic.

For peer review only

References

1. Arnoldi CC, Brodsky AE, Cauchoix J, et al. Lumbar spinal stenosis and nerve root entrapment syndromes. Definition and classification. Clin Orthop Relat Res 1976(115):4-5.
2. Ishimoto Y, Yoshimura N, Muraki S, et al. Associations between radiographic lumbar spinal stenosis and clinical symptoms in the general population: the Wakayama Spine Study. Osteoarthritis Cartilage 2013;**21**(6):783-8.
3. Deyo RA, Mirza SK. Trends and variations in the use of spine surgery. Clin Orthop Relat Res 2006;**443**:139-46.
4. Deyo RA, Mirza SK, Martin BI, et al. Trends, major medical complications, and charges associated with surgery for lumbar spinal stenosis in older adults. Jama 2010;**303**(13):1259-65.
5. Amundsen T, Weber H, Lilleas F, et al. Lumbar spinal stenosis. Clinical and radiologic features. Spine (Phila Pa 1976) 1995;**20**(10):1178-86.
6. Evans JG. Neurogenic Intermittent Claudication. Br Med J 1964;**2**(5415):985-7.
7. Fritz JM, Delitto A, Welch WC, et al. Lumbar spinal stenosis: a review of current concepts in evaluation, management, and outcome measurements. Arch Phys Med Rehabil 1998;**79**(6):700-8.
8. Ammendolia C, Stuber KJ, Rok E, et al. Nonoperative treatment for lumbar spinal stenosis with neurogenic claudication. Cochrane Database Syst Rev 2013(8):CD010712.
9. Malmivaara A, Slatis P, Heliovaara M, et al. Surgical or nonoperative treatment for lumbar spinal stenosis? A randomized controlled trial. Spine (Phila Pa 1976) 2007;**32**(1):1-8.
10. Weinstein JN, Tosteson TD, Lurie JD, et al. Surgical versus nonoperative treatment for lumbar spinal stenosis four-year results of the Spine Patient Outcomes Research Trial. Spine (Phila Pa 1976) 2010;**35**(14):1329-38.
11. Gibson JN, Waddell G. Surgery for degenerative lumbar spondylosis. Cochrane Database Syst Rev 2005(4):CD001352.
12. Fox MW, Onofrio BM, Hanssen AD. Clinical outcomes and radiological instability following decompressive lumbar laminectomy for degenerative spinal stenosis: a comparison of patients undergoing concomitant arthrodesis versus decompression alone. J Neurosurg 1996;**85**:793-802.
13. Johnsson KE, Willner S, Johnsson K. Postoperative instability after decompression for lumbar spinal stenosis. Spine 1986;**11**:107-10.
14. Tuite GF, Doran SE, Stern JD, et al. Outcome after laminectomy for lumbar spinal stenosis. Part II: Radiographic changes and clinical correlations. J Neurosurg 1994;**81**:707-15.
15. Thome C, Zevgaridis D, Leheta O, et al. Outcome after less-invasive decompression of lumbar spinal stenosis: a randomized comparison of unilateral laminotomy, bilateral laminotomy, and laminectomy. J Neurosurg Spine 2005;**3**(2):129-41.
16. Mayer HM. *Minimally Invasive Spine Surgery. A Surgical Manual*: Springer, 2005.
17. Oppenheimer JH, DeCastro I, McDonnell DE. Minimally invasive spine technology and minimally invasive spine surgery: a historical review. Neurosurg Focus 2009;**27**(3):E9.
18. Hu SS, Tribus CB, Diab M, et al. Spondylolisthesis and spondylolysis. J Bone Joint Surg Am 2008;**90**(3):656-71.
19. Meyerding HW. Spondylolisthesis. Surg Gynecol Obstet 1932;**54**:371-77.
20. Aryanpur J, Ducker T. Multilevel lumbar laminotomies: an alternative to laminectomy in the treatment of lumbar stenosis. Neurosurgery 1990;**26**(3):429-32; discussion 33.
21. Hutchinson PJ, Laing RJ, Waran V, et al. Assessing outcome in lumbar disc surgery using patient completed measures. Br J Neurosurg 2000;**14**(3):195-9.

For peer review only - http://bmjopen.bmj.com/site/about/guidelines.xhtml

22. Roland M, Morris R. A study of the natural history of low-back pain. Part II: development of guidelines for trials of treatment in primary care. Spine (Phila Pa 1976) 1983;**8**(2):145-50.
23. Roland M, Morris R. A study of the natural history of back pain. Part I: development of a reliable and sensitive measure of disability in low-back pain. Spine (Phila Pa 1976) 1983;**8**(2):141-4.
24. Brouwer S, Kuijer W, Dijkstra PU, et al. Reliability and stability of the Roland Morris Disability Questionnaire: intra class correlation and limits of agreement. Disabil Rehabil 2004;**26**(3):162-5.
25. Smeets R, Koke A, Lin CW, et al. Measures of function in low back pain/disorders: Low Back Pain Rating Scale (LBPRS), Oswestry Disability Index (ODI), Progressive Isoinertial Lifting Evaluation (PILE), Quebec Back Pain Disability Scale (QBPDS), and Roland-Morris Disability Questionnaire (RDQ). Arthritis Care Res (Hoboken) 2011;**63 Suppl 11**:S158-73.
26. Fairbank JC, Pynsent PB. The Oswestry Disability Index. Spine (Phila Pa 1976) 2000;**25**(22):2940-52; discussion 52.
27. van Hooff ML, Spruit M, Fairbank JC, et al. The Oswestry Disability Index (version 2.1a): validation of a Dutch language version. Spine (Phila Pa 1976) 2015;**40**(2):E83-90.
28. Williamson A, Hoggart B. Pain: a review of three commonly used pain rating scales. J Clin Nurs 2005;**14**(7):798-804.
29. Chapman JR, Norvell DC, Hermsmeyer JT, et al. Evaluating common outcomes for measuring treatment success for chronic low back pain. Spine (Phila Pa 1976) 2011;**36**(21 Suppl):S54-68.
30. Bombardier C. Outcome assessments in the evaluation of treatment of spinal disorders: summary and general recommendations. Spine (Phila Pa 1976) 2000;**25**(24):3100-3.
31. Gautschi OP, Corniola MV, Joswig H, et al. The timed up and go test for lumbar degenerative disc disease. J Clin Neurosci 2015;**22**(12):1943-8.
32. Gautschi OP, Smoll NR, Corniola MV, et al. Validity and Reliability of a Measurement of Objective Functional Impairment in Lumbar Degenerative Disc Disease: The Timed Up and Go (TUG) Test. Neurosurgery 2016;**79**(2):270-8.
33. Gautschi OP, Joswig H, Corniola MV, et al. Pre- and postoperative correlation of patient-reported outcome measures with standardized Timed Up and Go (TUG) test results in lumbar degenerative disc disease. Acta Neurochir (Wien) 2016;**158**(10):1875-81.
34. Victor ES, Marc LS. The five-repetition sit-to-stand test: evaluation of a simple and objective tool for the assessment of degenerative pathologies of the lumbar spine. 2018;**29**(4):380.
35. Jones SE, Kon SS, Canavan JL, et al. The five-repetition sit-to-stand test as a functional outcome measure in COPD. Thorax 2013;**68**(11):1015-20.
36. Niggemann P, Kuchta J, Grosskurth D, et al. Spondylolysis and isthmic spondylolisthesis: impact of vertebral hypoplasia on the use of the Meyerding classification. The British journal of radiology 2012;**85**(1012):358-62.
37. Herdman M, Gudex C, Lloyd A, et al. Development and preliminary testing of the new five-level version of EQ-5D (EQ-5D-5L). Qual Life Res 2011;**20**(10):1727-36.
38. M MV, K MV, S MAAE, et al. Dutch Tariff for the Five-Level Version of EQ-5D. Value Health 2016;**19**(4):343-52.
39. Chan AW, Tetzlaff JM, Altman DG, et al. SPIRIT 2013 statement: defining standard protocol items for clinical trials. Ann Intern Med 2013;**158**(3):200-7.
40. Jordan K, Dunn KM, Lewis M, et al. A minimal clinically important difference was derived for the Roland-Morris Disability Questionnaire for low back pain. J Clin Epidemiol 2006;**59**(1):45-52.
41. Versteegh M, Knies S, Brouwer W. From Good to Better: New Dutch Guidelines for Economic Evaluations in Healthcare. Pharmacoeconomics 2016;**34**(11):1071-74.
42. White IR, Royston P, Wood AM. Multiple imputation using chained equations: Issues and guidance for practice. Stat Med 2011;**30**(4):377-99.

For peer review only - http://bmjopen.bmj.com/site/about/guidelines.xhtml

43. Overdevest GM, Moojen WA, Arts MP, et al. Management of lumbar spinal stenosis: a survey among Dutch spine surgeons. Acta Neurochir (Wien) 2014;**156**(11):2139-45.
44. Soliman MAR, Ali A. Decompression of lumbar canal stenosis with a bilateral interlaminar versus classic laminectomy technique: a prospective randomized study. Neurosurg Focus 2019;**46**(5):E3.

AUTHOR'S CONTRIBUTION

J.R.J. Arjun Sharma: JAS

P.S. Gadjradj: PG

Wouter Mooijen: WM

Wilco Peul: WP

Maurits van Tulder: MvT

B.S. Harhangi: BH

JAS contributed to drafting article and took the lead in writing the manuscript. All authors provided critical feedback and helped shape the research and manuscript.

JAS, PG, WM, WP and BH devised the main conceptual ideas and designed the study.

MvT designed the statistical analysis and the economic evaluation.

PG and BH provided funding for conducting this study. JAS, PG, WM, WP, MvT and BH contributed to the study protocol.

For peer review only

APPENDIX

Informed consent materials/form

Interarcuaire decompressie versus laminectomie bij neurogene claudicatie.

- Ik heb de informatiebrief gelezen. Ook kon ik vragen stellen. Mijn vragen zijn voldoende beantwoord. Ik had genoeg tijd om te beslissen of ik meedoe.
- Ik weet dat meedoen vrijwillig is. Ook weet ik dat ik op ieder moment kan beslissen om toch niet mee te doen of te stoppen met het onderzoek. Daarvoor hoef ik geen reden te geven.
- Ik geef toestemming voor het informeren van mijn huisarts/de specialist die mij behandelt dat ik meedoe aan dit onderzoek.
- Ik geef toestemming voor het opvragen van informatie bij de specialist die mij behandelt over de wervelkanaalstenose.
- Ik geef toestemming voor het verzamelen en gebruiken van mijn gegevens voor de beantwoording van de onderzoeksvraag in dit onderzoek.
- Ik weet dat voor de controle van het onderzoek sommige mensen toegang tot al mijn gegevens kunnen krijgen. Die mensen staan vermeld in deze informatiebrief. Ik geef toestemming voor die inzage door deze personen.

<voor zover van toepassing:>

- Ik geef toestemming voor het informeren van mijn huisarts en/of behandelend specialist van onverwachte bevindingen die van belang (kunnen) zijn voor mijn gezondheid.
- Ik geef □ **wel**
□ **geen**
toestemming om mijn persoonsgegevens langer te bewaren en te gebruiken voor toekomstig onderzoek op het gebied van mijn aandoening en de onderzochte behandelwijze
- Ik geef □ **wel**
□ **geen**
toestemming om mij na dit onderzoek opnieuw te benaderen voor een vervolgonderzoek.
- Ik wil □ **wel**
□ **niet**
geïnformeerd worden over welke behandeling ik heb gehad/in welke groep ik zat.

- Ik wil meedoen aan dit onderzoek.

For peer review only

Naam proefpersoon:

Handtekening: Datum : __ / __ / __

Ik verklaar dat ik deze proefpersoon volledig heb geïnformeerd over het genoemde onderzoek.

Als er tijdens het onderzoek informatie bekend wordt die de toestemming van de proefpersoon zou kunnen beïnvloeden, dan breng ik hem/haar daarvan tijdig op de hoogte.

Naam onderzoeker (of diens vertegenwoordiger):

Handtekening: Datum: __ / __ / __

Aanvullende informatie is gegeven door:

Naam:

Functie:

Handtekening: Datum: __ / __ / __

----

* Doorhalen wat niet van toepassing is.

For peer review only - http://bmjopen.bmj.com/site/about/guidelines.xhtml

Reporting checklist for protocol of a clinical trial.

Based on the SPIRIT guidelines.

Instructions to authors

Complete this checklist by entering the page numbers from your manuscript where readers will find each of the items listed below.

Your article may not currently address all the items on the checklist. Please modify your text to include the missing information. If you are certain that an item does not apply, please write "n/a" and provide a short explanation.

Upload your completed checklist as an extra file when you submit to a journal.

In your methods section, say that you used the SPIRITreporting guidelines, and cite them as:

Chan A-W, Tetzlaff JM, Altman DG, Laupacis A, Gøtzsche PC, Krleža-Jerić K, Hróbjartsson A, Mann H, Dickersin K, Berlin J, Doré C, Parulekar W, Summerskill W, Groves T, Schulz K, Sox H, Rockhold FW, Rennie D, Moher D. SPIRIT 2013 Statement: Defining standard protocol items for clinical trials. Ann Intern Med. 2013;158(3):200-207

		Reporting Item	Page Number
**Administrative information**			
Title	#1	Descriptive title identifying the study design, population, interventions, and, if applicable, trial acronym	1

For peer review only

Trial registration	#2a	Trial identifier and registry name. If not yet registered, name of intended registry	1
Trial registration: data set	#2b	All items from the World Health Organization Trial Registration Data Set	1
Protocol version	#3	Date and version identifier	1
Funding	#4	Sources and types of financial, material, and other support	1
Roles and responsibilities: contributorship	#5a	Names, affiliations, and roles of protocol contributors	1 and 24
Roles and responsibilities: sponsor contact information	#5b	Name and contact information for the trial sponsor	1
Roles and responsibilities: sponsor and funder	#5c	Role of study sponsor and funders, if any, in study design; collection, management, analysis, and interpretation of data; writing of the report; and the decision to submit the report for publication, including whether they will have ultimate authority over any of these activities	1
Roles and responsibilities: committees	#5d	Composition, roles, and responsibilities of the coordinating centre, steering committee, endpoint adjudication committee, data management team, and	n/a

For peer review only

		other individuals or groups overseeing the trial, if applicable (see Item 21a for data monitoring committee)	
**Introduction**			
Background and rationale	#6a	Description of research question and justification for undertaking the trial, including summary of relevant studies (published and unpublished) examining benefits and harms for each intervention	5
Background and rationale: choice of comparators	#6b	Explanation for choice of comparators	5-6
Objectives	#7	Specific objectives or hypotheses	6
Trial design	#8	Description of trial design including type of trial (eg, parallel group, crossover, factorial, single group), allocation ratio, and framework (eg, superiority, equivalence, non-inferiority, exploratory)	6
**Methods: Participants, interventions, and outcomes**			
Study setting	#9	Description of study settings (eg, community clinic, academic hospital) and list of countries where data will be collected. Reference to where list of study sites can be obtained	7

For peer review only

Eligibility criteria	#10	Inclusion and exclusion criteria for participants. If applicable, eligibility criteria for study centres and individuals who will perform the interventions (eg, surgeons, psychotherapists)	7-8
Interventions: description	#11a	Interventions for each group with sufficient detail to allow replication, including how and when they will be administered	8-9
Interventions: modifications	#11b	Criteria for discontinuing or modifying allocated interventions for a given trial participant (eg, drug dose change in response to harms, participant request, or improving / worsening disease)	n/a
Interventions: adherance	#11c	Strategies to improve adherence to intervention protocols, and any procedures for monitoring adherence (eg, drug tablet return; laboratory tests)	n/a
Interventions: concomitant care	#11d	Relevant concomitant care and interventions that are permitted or prohibited during the trial	9
Outcomes	#12	Primary, secondary, and other outcomes, including the specific measurement variable (eg, systolic blood pressure), analysis metric (eg, change from baseline, final value, time to event), method of aggregation (eg, median, proportion), and time point for each outcome. Explanation of the clinical relevance of chosen efficacy and harm outcomes is strongly recommended	9-14

For peer review only

Participant timeline	#13	Time schedule of enrolment, interventions (including any run-ins and washouts), assessments, and visits for participants. A schematic diagram is highly recommended (see Figure)	15
Sample size	#14	Estimated number of participants needed to achieve study objectives and how it was determined, including clinical and statistical assumptions supporting any sample size calculations	16
Recruitment	#15	Strategies for achieving adequate participant enrolment to reach target sample size	n/a
**Methods: Assignment of interventions (for controlled trials)**			
Allocation: sequence generation	#16a	Method of generating the allocation sequence (eg, computer-generated random numbers), and list of any factors for stratification. To reduce predictability of a random sequence, details of any planned restriction (eg, blocking) should be provided in a separate document that is unavailable to those who enrol participants or assign interventions	16
Allocation concealment mechanism	#16b	Mechanism of implementing the allocation sequence (eg, central telephone; sequentially numbered, opaque,	16

For peer review only

		sealed envelopes), describing any steps to conceal the sequence until interventions are assigned	
Allocation: implementation	#16c	Who will generate the allocation sequence, who will enrol participants, and who will assign participants to interventions	16
Blinding (masking)	#17a	Who will be blinded after assignment to interventions (eg, trial participants, care providers, outcome assessors, data analysts), and how	16
Blinding (masking): emergency unblinding	#17b	If blinded, circumstances under which unblinding is permissible, and procedure for revealing a participant's allocated intervention during the trial	16
**Methods: Data collection, management, and analysis**			
Data collection plan	#18a	Plans for assessment and collection of outcome, baseline, and other trial data, including any related processes to promote data quality (eg, duplicate measurements, training of assessors) and a description of study instruments (eg, questionnaires, laboratory tests) along with their reliability and validity, if known. Reference to where data collection forms can be found, if not in the protocol	16-17

For peer review only

Data collection plan: retention	#18b	Plans to promote participant retention and complete follow-up, including list of any outcome data to be collected for participants who discontinue or deviate from intervention protocols	17
Data management	#19	Plans for data entry, coding, security, and storage, including any related processes to promote data quality (eg, double data entry; range checks for data values). Reference to where details of data management procedures can be found, if not in the protocol	17
Statistics: outcomes	#20a	Statistical methods for analysing primary and secondary outcomes. Reference to where other details of the statistical analysis plan can be found, if not in the protocol	17-19
Statistics: additional analyses	#20b	Methods for any additional analyses (eg, subgroup and adjusted analyses)	17-19
Statistics: analysis population and missing data	#20c	Definition of analysis population relating to protocol non-adherence (eg, as randomised analysis), and any statistical methods to handle missing data (eg, multiple imputation)	18
**Methods: Monitoring**			
Data monitoring: formal committee	#21a	Composition of data monitoring committee (DMC); summary of its role and reporting structure; statement of whether it is independent from the sponsor and competing interests; and reference to where further	19

For peer review only

		details about its charter can be found, if not in the protocol. Alternatively, an explanation of why a DMC is not needed	
Data monitoring: interim analysis	#21b	Description of any interim analyses and stopping guidelines, including who will have access to these interim results and make the final decision to terminate the trial	19
Harms	#22	Plans for collecting, assessing, reporting, and managing solicited and spontaneously reported adverse events and other unintended effects of trial interventions or trial conduct	20
Auditing	#23	Frequency and procedures for auditing trial conduct, if any, and whether the process will be independent from investigators and the sponsor	n/a
**Ethics and dissemination**			
Research ethics approval	#24	Plans for seeking research ethics committee / institutional review board (REC / IRB) approval	19
Protocol amendments	#25	Plans for communicating important protocol modifications (eg, changes to eligibility criteria, outcomes, analyses) to relevant parties (eg, investigators, REC / IRBs, trial participants, trial registries, journals, regulators)	19

For peer review only

Consent or assent	#26a	Who will obtain informed consent or assent from potential trial participants or authorised surrogates, and how (see Item 32)	20
Consent or assent: ancillary studies	#26b	Additional consent provisions for collection and use of participant data and biological specimens in ancillary studies, if applicable	n/a
Confidentiality	#27	How personal information about potential and enrolled participants will be collected, shared, and maintained in order to protect confidentiality before, during, and after the trial	20
Declaration of interests	#28	Financial and other competing interests for principal investigators for the overall trial and each study site	1 (n/a)
Data access	#29	Statement of who will have access to the final trial dataset, and disclosure of contractual agreements that limit such access for investigators	20
Ancillary and post trial care	#30	Provisions, if any, for ancillary and post-trial care, and for compensation to those who suffer harm from trial participation	20
Dissemination policy: trial results	#31a	Plans for investigators and sponsor to communicate trial results to participants, healthcare professionals, the public, and other relevant groups (eg, via publication, reporting in results databases, or other data sharing arrangements), including any publication restrictions	20

For peer review only

Dissemination policy: authorship	#31b	Authorship eligibility guidelines and any intended use of professional writers	n/a
Dissemination policy: reproducible research	#31c	Plans, if any, for granting public access to the full protocol, participant-level dataset, and statistical code	n/a
**Appendices**			
Informed consent materials	#32	Model consent form and other related documentation given to participants and authorised surrogates	25-26
Biological specimens	#33	Plans for collection, laboratory evaluation, and storage of biological specimens for genetic or molecular analysis in the current trial and for future use in ancillary studies, if applicable	n/a

The SPIRIT checklist is distributed under the terms of the Creative Commons Attribution License CC-BY-ND 3.0. This checklist was completed on 05. January 2020 using https://www.goodreports.org/, a tool made by the EQUATOR Network in collaboration with Penelope.ai

For peer review only

BMJ Open

SIZE study: study protocol of a multi-centre, randomised controlled trial to compare the effectiveness of an interarcuair decompression versus extended decompression in patients with intermittent neurogenic claudication caused by lumbar spinal stenosis.

Journal:	*BMJ Open*
Manuscript ID	bmjopen-2020-036818.R1
Article Type:	Protocol
Date Submitted by the Author:	01-Jul-2020
Complete List of Authors:	Arjun Sharma, Jamie; Erasmus Medical Center, Neurosurgery<br>Gadjradj, Pravesh; Erasmusmc, Neurosurgery<br>Peul, Wilco; Leiden University Medical Center, Neurosurgery<br>van Tulder, Maurits; University of Amsterdam, Health Sciences<br>Moojen, Wouter; Medical Centre Haaglanden, Neurosurgery; Leiden University Medical Center, Neurosurgery<br>Harhangi, Biswadjiet; Erasmus MC, ; Erasmus Medical Center, Neurosurgery
**Primary Subject Heading**:	Surgery
Secondary Subject Heading:	Surgery
Keywords:	NEUROSURGERY, Spine < ORTHOPAEDIC & TRAUMA SURGERY, Neurosurgery < SURGERY

SCHOLARONE™
Manuscripts

I, the Submitting Author has the right to grant and does grant on behalf of all authors of the Work (as defined in the below author licence), an exclusive licence and/or a non-exclusive licence for contributions from authors who are: i) UK Crown employees; ii) where BMJ has agreed a CC-BY licence shall apply, and/or iii) in accordance with the terms applicable for US Federal Government officers or employees acting as part of their official duties; on a worldwide, perpetual, irrevocable, royalty-free basis to BMJ Publishing Group Ltd ("BMJ") its licensees and where the relevant Journal is co-owned by BMJ to the co-owners of the Journal, to publish the Work in this journal and any other BMJ products and to exploit all rights, as set out in our licence.

The Submitting Author accepts and understands that any supply made under these terms is made by BMJ to the Submitting Author unless you are acting as an employee on behalf of your employer or a postgraduate student of an affiliated institution which is paying any applicable article publishing charge ("APC") for Open Access articles. Where the Submitting Author wishes to make the Work available on an Open Access basis (and intends to pay the relevant APC), the terms of reuse of such Open Access shall be governed by a Creative Commons licence – details of these licences and which Creative Commons licence will apply to this Work are set out in our licence referred to above.

Other than as permitted in any relevant BMJ Author's Self Archiving Policies, I confirm this Work has not been accepted for publication elsewhere, is not being considered for publication elsewhere and does not duplicate material already published. I confirm all authors consent to publication of this Work and authorise the granting of this licence.

For peer review only

SIZE study: study protocol of a multi-centre, randomised controlled trial to compare the effectiveness of an interarcuair decompression versus extended decompression in patients with intermittent neurogenic claudication caused by lumbar spinal stenosis.

Article type: design article

J.R.J. Arjun Sharma, BSc[1] , P.S. Gadjradj MD[2], W.C. Peul MD, PhD[2], MBA, M.W. van Tulder PhD[3], W.A. Moojen MD, PhD, MSc[2] , B.S. Harhangi MD, PhD, MSc[1] for the SIZE-study group

1. Department of Neurosurgery, Erasmus MC, University Medical Centre, Rotterdam, The Netherlands
2. Department of Neurosurgery, Haaglanden Medical Centre & Leiden University Medical Centre and Haga Teaching Hospital, The Netherlands
3. Department of Health Sciences, Faculty of Science, Vrije Universiteit Amsterdam, MOVE research institute Amsterdam, Amsterdam, the Netherlands.

Protocol version: 3
Funding sources/sponsor: This work was supported by MRace grant, number 2017-17106 (Institutional Grant of Erasmus Medical Centre)
IRB approval status: MEC- 2018-093
Trial registration/ClinicalTrials.gov: NCT03480893
Conflicts of Interest: None declared.

Abstract word count: 269
Manuscript word count: 4083 [excluding references, figures, and tables]
References: 51
Figures: 0
Tables: 1
Supplementary material: 0
Keywords: Spinal Stenosis/surgery, Decompression, Lumbar Vertebrae/surgery, Humans, Laminectomy
Running title: The SIZE study.

Acknowledgements: the SIZE-study group consists, except for the authors, out of
Drs. H.P. van Putten
Dr. R. van Rolde

The authors would furthermore gratefully acknowledge Katya Mauff for statistical advice.

#Corresponding author:
B.S. Harhangi MD, PhD, MSc
Department of Neurosurgery
Erasmus Medical Centre, Rotterdam, the Netherlands
Dr. Molewaterplein 40, 3015 GD
Postbus 2040, 3000 CA Rotterdam
b.s.harhangi@erasmusmc.nl

For peer review only - http://bmjopen.bmj.com/site/about/guidelines.xhtml

Abbreviations:

LSS: Lumbar Spinal Stenosis

INC: Intermittent Neurogenic Claudication

MRI: Magnetic Resonance Imaging

ODI: Oswestry Disability Index

NRS: Numeric Rating Scale

RMDQ: Roland Morris Disability Questionnaire

TUG: Timed-Up and Go Test

6MWT: 6-Minute Walking Test

For peer review only

For peer review only - http://bmjopen.bmj.com/site/about/guidelines.xhtml

ABSTRACT

Introduction

Intermittent neurogenic claudication (INC) is often caused by lumbar spinal stenosis (LSS). Laminectomy is considered a frequently used surgical technique for LSS. Previous studies have shown that laminectomy can potentially cause lumbar instability. Less invasive techniques, preserving midline structures including the bilateral small size interarcuair decompression, are currently applied. Due to lack of evidence and consensus, surgeons have to rely on their training and own experiences to choose the best surgical techniques for their patients. Hence an observer and patient blinded multi-centre, randomised controlled trial was designed to determine the effectiveness and cost-effectiveness of bilateral interarcuair decompression versus laminectomy for LSS.

Methods and analysis

174 patients above 40 years with at least 12 weeks of complaints of INC will be recruited. Patients are eligible for inclusion if they have a clinical indication for surgery for INC with a MRI showing signs of LSS. Patients will be randomised to laminectomy or bilateral interarcuair decompression. The primary outcome is functional status measured with the Roland Morris Disability Questionnaire at 12 months. Secondary outcomes consist of pain intensity, self-perceived recovery, functional status measured with the Oswestry Disability Index, and a physical examination. Outcome measurement moments will be scheduled at 3 and 6 weeks, and at 3, 6, 12, 18, 24, 36 and 48 months after surgery. Physical examination will be performed at 6 weeks, and 12, 24 and 48 months. An economic evaluation will be performed and questionnaires will be used to collect cost data.

Ethics and dissemination

The Medical Ethical Committee of the Erasmus Medical Centre Rotterdam approved this study: NL.65826.078.18. the results will be published in an international peer-reviewed journal.

For peer review only - http://bmjopen.bmj.com/site/about/guidelines.xhtml

ARTICLE SUMMARY

'Strengths and limitations of this study'

- Patient and observer blinded randomised controlled design.
- Both patient-reported outcomes and objective functional test outcomes will be measured.
- Inclusion of an economic evaluation.
- Instability as outcome may not be measurable in two years of follow-up.
- Diversity in terminology on posterior decompression techniques may lead to variability on both surgical techniques.

For peer review only

For peer review only - http://bmjopen.bmj.com/site/about/guidelines.xhtml

INTRODUCTION

Lumbar spinal stenosis (LSS) is described as the narrowing of the diameter of the spinal and nerve root canals, which can cause compression of the neural structures in the canals[1]. LSS is a common medical condition in the aging population and is considered the most common reason for lumbar spine surgery for people over the age of 65[2-4]. Intermittent neurogenic claudication (INC) is the most important symptom of LSS, with complaints of leg pain and heaviness of the legs, usually bilateral, which exacerbate during standing and walking. Apart from leg pain, back pain is often also present[5]. As the symptoms aggravate, the ability to walk can become considerably limited, which is the dominant functional impairment and the most common reason for seeking medical care[6 7].

Conservative treatment, such as physical therapy and pain medication, is the first treatment option and may give some relief of symptoms[8]. Previous research may show that there is sparse evidence of the effectiveness of surgery compared to non-operative treatment[9]. However, usually when conservative treatment fails, surgical treatment is considered the next option[10 11]. The first technique described to widen the lumbar spinal canal is the laminectomy technique, also known as wide bony decompression , which is a widely used surgical technique for patients with INC caused by LSS[12]. Given that patients often experience post-operative back pain after laminectomy, it is hypothesized that laminectomy is a ground for potential lumbar instability and iatrogenic scoliosis[13-15]. Performing a procedure with potentially more complications in an elderly population could be considered as doubtful. Hence, less invasive techniques, such as the bilateral small size interarcuair decompression, otherwise known as a bilateral laminotomy, limited bony decompression or interlaminar decompression, were developed and implemented in clinical practice. These techniques require less bone tissue to be removed, which possibly can reduce the prevalence of lumbar instability in these patients and consequently improve functioning[16]. Furthermore, it is believed that, compared to laminectomy, these less invasive techniques

For peer review only - http://bmjopen.bmj.com/site/about/guidelines.xhtml

have the ability to decompress the nerves while preserving the spinal integrity. However, the risk of an insufficient decompression may be higher, possibly resulting in reoperations due to residual stenosis[16-18]. The presumption is often made that, after a wide decompression, recurrence of the complaints are scarce. The opinions on this matter are diverse and subject to debate.[19]. Therefore, a randomised controlled trial is needed to clarify whether bilateral interarcuair decompression is effective and cost-effective compared to a classical laminectomy.

Study objectives

The primary objective of this study is to determine whether bilateral interarcuair decompression is more effective than laminectomy in patients with INC caused by LSS. The secondary objective is to evaluate if bilateral interarcuair decompression is more cost-effective than laminectomy.

Study design

The study is designed as a multi-centre, patient and observer blinded randomised controlled trial with an economic evaluation alongside. Participants will be allocated to one of the two groups: laminectomy or interarcuair decompression.

For peer review only

METHODS

Study setting/population

In total 174 patients with a MRI-confirmed LSS will be recruited. All patients who will be included should have an indication for surgery due to LSS with minimally 12 weeks of complaints of INC and failed conservative treatment. Patients will be recruited from two hospitals and one private health clinic in the Netherlands:

- Erasmus Medical Centre in Rotterdam.
- Maasstad Hospital in Rotterdam.
- Park Medical Centre in Rotterdam.

Eligibility criteria

In order to be suitable to participate in this study, patients must meet all of the following criteria:

Inclusion criteria

- At least 12 weeks of complaints of INC with a leg pain level above 30 on the Visual Analogue Scale, without sufficient response to conservative management.
- Indication for an operation according to consensus of surgeon
- MRI demonstrating LSS
- Age above 40 years
- Sufficient knowledge of the Dutch language in order to comprehend the questionnaires and patient information
- Written informed consent

Exclusion criteria

For peer review only - http://bmjopen.bmj.com/site/about/guidelines.xhtml

148 Patients who meet any of the following criteria will be excluded from participating in this study.

149 - History of lumbar spine surgery

150 - More than 2 lumbar levels needing surgery

151 - Degenerative spondylolisthesis greater than Meyerding grade I (on a scale of I to V)[20 21]

152 - Concomitant scoliosis or disc herniation

153 - Severe comorbid medical disorder (American Society of Anesthesiologists > 3)

154 - Serious psychopathologic disorder

155 - Pregnancy

156 - Active malignancy

157 - Plans to move abroad during study period.

158 Eligible patients will be randomised in a 1:1 ratio to the two groups.

159 **Interventions**

160 ***Intervention: bilateral small size interarcuair decompression***

161 The intervention consists of the interarcuair decompression, otherwise known as a bilateral
162 laminotomy, limited bony decompression or an interlaminar decompression. General anaesthesia is to
163 be administered. A midline skin incision is made, after which the paravertebral muscles are dissected
164 subperiosteally and retracted bilaterally. Decompression will be applied through decompression of the
165 ligamentum flavum and partial laminotomy if necessary. The lateral recess will be opened bilaterally and
166 a partial medial facetectomy will be performed to decompress neuronal structures including the exciting
167 nerve root.. The lamina will be removed up to the ligamentum flavum. Interspinous ligaments are
168 spared.[22] The wound is closed in layers. Patients are operated with a loupe magnification or microscope
169 depending on the surgeon's preference.

For peer review only - http://bmjopen.bmj.com/site/about/guidelines.xhtml

Usual care: conventional laminectomy

Usual care consists of conventional laminectomy, otherwise known as wide bony decompression. General anaesthesia is to be administered. A midline skin incision is made over the spinous processes. The laminae of the affected level(s) are exposed subperiosteally, and the supraspinous ligament is incised. The spinous process is removed. The supra and interspinous ligament of the affected level is removed by drill or Kerrison punched. Both laminae are removed at the affected level, leaving the facet joint intact. The lateral recess is opened bilaterally and a partial medial facetectomy will be performed to decompress neuronal structures including the exciting nerve root.[16]. To clarify, when a single level stenosis is present (e.g. L4-L5), both laminae L4 and L5 will be removed. When a double level stenosis (e.g. L3-L4 and L4-L5) is present, three laminae (L3, L4 and L5) will be removed. The wound is closed in layers with or without a suction drain. Patients will be operated with loupe magnification or microscope depending on the surgeon's preference.

Use of co-intervention

Pain medication will be provided to patients after surgery, should this be necessary. Further, the use of co-interventions will be tracked by cost questionnaires, in which medication usage and any health care utilization is monitored throughout the follow-up period.

Outcomes

Primary outcomes

The primary outcome is a functional status measured using the Roland-Morris Disability Questionnaire (RMDQ). This is a 24-point questionnaire that is designed to assess the functional status in patients with low back pain. This outcome measure has been identified as one of the most commonly used outcomes in a population with chronic low back pain[23-27].

For peer review only - http://bmjopen.bmj.com/site/about/guidelines.xhtml

Secondary outcomes

Oswestry Disability Index (ODI)

The ODI is one of the principal condition-specific outcome measures used in de management of spinal disorders[28]. The current version 2.1a will be used[29]. It consists of 10 questions, each with six possible answers and each answer option receives a score of 0 to 5 points, yielding a score range between 0 and 50, which is scaled to a 100% range. The questions focus on a range of daily physical functions and how the back or leg pain is affecting the patient's ability to manage in everyday life.

Numeric Rating Scale for leg and low back pain

This parameter will measure the experienced pain intensity in both legs (affected and non-affected) and back. Pain intensity will be rated on an 11-point scale, varying from 0 representing 'no pain' to 10 'the most terrible pain imaginable'. Patients are not permitted to see the pain score indicated during previous visits. Reliability and validity of NRS have been shown[30].

Short Form 36 (SF-36)

The quality of life will be measured with the Dutch version of the Short Form 36 (SF36). The SF36 has been applied and successfully validated in populations with low back pain[31]. This questionnaire relates to the analysis of the general functional status of the patient. The questionnaire is divided into eight health concepts: (1) physical functioning, (2) role limitations because of physical health problems, (3) bodily pain, (4) social functioning, (5) general mental health (psychological distress and well-being); (6) emotional role limitations; (7) vitality and (8) general health perceptions. The scores of the different health concepts are added up into a scale of 0-100. A higher score reflects a better general health condition. The physical and mental health will be assessed separately.

For peer review only - http://bmjopen.bmj.com/site/about/guidelines.xhtml

Patients' perceived recovery

Recovery will be measured using a seven-point Likert scale. The score on this scale varies from 'complete recovery' to 'worse than ever'. The outcomes 'complete recovery and 'almost complete recovery' will be dichotomized and considered as recovered[32].

Patients' satisfaction

Patients' satisfaction of change and satisfaction of treatment will be assessed using a seven-point Likert scale, ranging from 'completely satisfied' to 'completely dissatisfied'. 'Completely satisfied' and 'almost completely satisfied' will be defined as good outcome.

Physical examination

A physical examination will be performed before surgery, at 6 weeks, 12, 24 and 48 months after surgery. This examination will include a brief neurologic examination, the Timed Up and Go Test (TUG), the Timed Chair-Stand Test, the Six-Minute Walking Test (6MWT) and patient's weight.

a. *Neurologic examination*

The neurologic examination will include the muscle strength of the quadriceps, the iliopsoas, the hamstrings, the gastrocnemicus and the tibialis anterior muscle, which will be noted as a difference in strength between both legs. We will evaluate if there is a difference in muscle strength between the affected leg and the other leg.

b. *Timed Up and Go Test (TUG)*

Patients are asked to sit in a chair with their arms resting on the armrests. On direction of the research nurse, patients have to walk as fast as possible (without running) to a wall at 3-m distance of the chair. When they have reached that wall, a turn of 180 degrees is made and they walk directly back to the chair and sit down. The research nurse will measure the time (in seconds) it takes for a patient, to perform this action. The time is stopped when the patients

For peer review only - http://bmjopen.bmj.com/site/about/guidelines.xhtml

are seated again[33]. Patients need to perform this test, three times. Patients are allowed to wear their regular shoes and use a walking aid, if necessary. The TUG test has been described as a valid, reliable tool in the assessment of objective functional impairment in patients with lumbar degenerative disc disease[34 35]. However, this has not been done for LSS. The validation for LSS will be conducted in this study.

c. *Timed Chair-Stand Test*

Patients are asked to sit down on an armless chair, firmly placed against a wall, with their arms folded across their chest. They are instructed to place their feet flat on the ground. To become familiarized with the exercise, the patients are asked to stand up and sit down again without using their arms. If assistance is required during this action, or this movement cannot be completed by the patient, the test is abandoned. If the manoeuvre is possible, the patient will be asked to, on the word "go", stand up entirely and sit down again, 5 times as quick as possible. The research nurse will time the 5 movements in seconds from the command "go" to the completed fifth stand. The measured time will be noted as the score. If the patient is unable to perform the test 5 times, a maximum score will be noted of 30 seconds[36 37].

d. *Six-Minute Walking Test (6MWT)*

This test is performed to assess the gait pattern, walking speed and physical endurance of patients. The patient will be requested to walk at such a speed that at the end of 6 minutes, the patient will have the feeling of maximum output[38]. Before taking the test, the patient will be given the instructions to install the 6MWT application on their cell phone[39 40]. During these 6 minutes this application will measure the walking distance. During the walking test, the patient is permitted to use a walking aid and/or orthosis which will be noted.

Functional lumbar X-rays

For peer review only - http://bmjopen.bmj.com/site/about/guidelines.xhtml

Functional X-rays will be obtained from all patients. The X-ray will be made standing from AP and lateral position to assess spondylolisthesis. X-rays will also be made in maximum flexion and extension position. Degenerative spondylolisthesis is defined as a vertebral slip of at least 3 mm[41]. After the measurement of the vertebral slip, this will be classified according to the Meyerding classification. This will be obtained at 6 weeks postoperatively, for level verification and for verification of correct procedure and after two years postoperatively to look for possible instability.

EuroQol-5-dimension- 5-levels

The EuroQol (EQ-5D-5L) is a validated instrument to assess health-related quality of life (QOL). The tool measures five dimensions:

- Mobility
- Self-care
- Pain/discomfort
- Daily activities
- Anxiety

Each dimension consists of one item, in which five levels are distinguished (no problems, minor problems, moderate problems, major problems, severe problems, unable to). The Dutch tariff of the EQ-5D-5L will be used to calculate the quality-adjusted life years (QALYs)[42 43].

<u>Cost-diaries</u>

Based on a diary with made costs, which will be completed by patients, insight will be obtained in the need for care, working participation and direct- and indirect medical cost. Direct medical cost will be estimated on basis of the cost centre method. In addition it is expected that the study population will consist of elderly people who may be retired. For the cost calculation additional costs, such as help in house, transport, help from family etc. will be taken in account.

For peer review only

For peer review only - http://bmjopen.bmj.com/site/about/guidelines.xhtml

285 For an estimation of direct- and indirect cost permission of the patient will be asked to request the total amount of cost incurred during the research period from the insurance company. No consideration by participation in the investigation as compared to daily practice will be deducted. The additional investigational costs include mainly the additional visits to the outpatient clinic.

289 The patient will be requested to keep a diary for the financial aspects of the consequences of INC and corresponding treatment. The patient will be requested to record the following items:

291 - Visits to GP
292 - Visits to physiotherapist
293 - Visits to specialist
294 - Alternative medicines and devices (e.g. rollator, etc.)
295 - Number of days of hospitalization
296 - Pain medication; dosage and frequency
297 - Illness-related days of absence at work, if any
298 - Cost of loss production and substitute manpower, if any
299 - Additional travelling expenses on account of INC
300 - Help in house holding

302 *Complication and re-operation incidence*

303 Immediately after surgery, the surgeon will report any perioperative complications such as cerebrospinal fluid leakage, nerve root damage and if the operation was implemented at the wrong vertebral level. Additionally, a systematic assessment of complications (including urinary tract infection, progressive neurological deficit and secondary bleeding) will be carried out by the surgeon and the research nurse shortly after discharge.

308 Reoperation is considered a bad outcome. The incidence of reoperation as well as perioperative morbidity will be assessed in both groups, using surgical parameters such as blood loss, time of surgery and length of hospital stay.

311 We used the SPIRIT checklist when writing our report[44].

For peer review only - http://bmjopen.bmj.com/site/about/guidelines.xhtml

Patient and public involvement

No patients involved.

For peer review only

For peer review only - http://bmjopen.bmj.com/site/about/guidelines.xhtml

Table 1: Participant timeline schedule

Table	Visit plan and case report forms											
	Intake	Surgery (t_0)	1-2 day(s) following surgery	FU 3 weeks	FU 6 weeks	FU 3 months	FU 6 months	FU 12 months	FU 18 months	FU 24 months	FU 36 months	FU 48 months
Visit surgeon	x				x							
Research nurse visit	x				x			x		x		x
Randomisation		X										
Surgery		X										
Discharge			x									
Physical examination	x				x			x		x		x
Functional x-rays	x				x					x		
Roland Morris Questionnaire	x			x	x	x	x	x		x	x	x
Oswestry Disability Index (ODI)	x			x	x	x	x	x		x	x	x
NRS leg and back pain	x			x	x	x	x	x		x	x	x
Patient self-received recovery and satisfaction	x			x	x	x	x	x		x	x	x
EuroQol-5D	x			x	x	x	x	x		x	x	x
Cost questionnaires	x			x	x	x	x	x	x	x		
Short Form 36 (SF-36)	x			x	x	x	x	x		x	x	x
Revisit and complications	WITH OCCURRENCE											

For peer review only

Sample Size

The sample size of this study is calculated for the primary endpoint of group difference in the RMDQ at 12 months. According to literature, results obtained after interarcuair decompression should be 3 points lower on the RMDQ compared to those for the laminectomy group in order to detect superiority[45].

Hence the sample size of this study is based on a superiority design, using a delta of 3, an assumed true mean difference of 0 and a pooled standard deviation of 6. Using an alpha of 0.05, and a power of 90%, we calculated a sample size of 69 patients per group. Accounting for a 20% loss in follow-up, we will therefore enrol 174 patients (87 patients per group).

Allocation

Computerized block randomisation will take place after the patient has been anaesthetised. Allocation concealment will be assured by using ALEA, a web-based data system supervised by the Clinical Trial Centre (CTC) of the Erasmus MC. Variable block sizes of 4, 6 and 8 will be used and stratified by treatment centre. Patients will be randomised in a 1:1 ratio to laminectomy or bilateral interarcuair decompression. Each patient will be given a unique study number.

Blinding

All researchers and patients will be blinded for the treatment. This is possible, because there are no fundamental differences between both procedures (e.g. general anaesthesia, spinal incision, inpatient procedure). The statistical analyses will be performed blinded. The surgeon cannot be blinded.

Data collection methods

For peer review only - http://bmjopen.bmj.com/site/about/guidelines.xhtml

For peer review only

All patient reported outcomes will be collected preoperatively and after 3 weeks, 6 weeks, 3 months, 6 months, 12 months, 18 months, 24 months, 36 months and 48 months (see Table 1). Patients will receive an email with a link, reminding them of the upcoming data collection and requesting them to fill in the web-based questionnaires. If the questionnaires are not completed, the patient will receive a reminder after one and two weeks. After two weeks, non-responders will be contacted by phone.

Data management

Data from initial visits to the hospital, follow-up visits and questionnaires will be entered into a database via an electronic data system (Gemstracker). This data will be noted and analysed by using coded information (study numbers) without any personal identifiers. Data will be stored via the secure data management system of the trial coordination centre of the Erasmus MC.

Statistical analysis

Patient characteristics will be summarized per treatment group to determine whether the randomisation was balanced. Continuous variables will be presented using means and standard deviations, or in the case of non-normally distributed variables using medians and interquartile ranges. Normality will be graphically assessed. Categorical variables will be presented using counts and percentages. The primary analysis will study differences in the primary outcome measure (RMDQ). The primary endpoint of interest is 12 months after randomisation. The results of the RMDQ will be assessed using generalized linear mixed models (GLMM), to account for the repeated measurements within patients and the multi-centre stratification.

The secondary outcomes (ODI, NRS leg and back pain score, TUG test, TCST and the SF-36) will be similarly assessed using GLMMs. In all analyses the primary assessment of treatment effect will be the estimate of the main effect within the appropriate model at 12 months, adjusted for the stratification

For peer review only

For peer review only - http://bmjopen.bmj.com/site/about/guidelines.xhtml

factors and main covariates. Time to recovery will be analysed using survival analysis (Cox proportional hazards). At 24, 36 and 48 months, the GLMM repeated-measurement analyses using the compound symmetry covariance structure will be used while *group* (interarcuair and conventional), *time* (moments of measurement) and the *interaction* between Group and Time (Group x Time) will be entered as independent variables. Dependent variables are the primary and secondary outcome parameters. Likert scales for self-reported patient recovery and satisfaction will be assessed using descriptive statistics (counts and percentages) and appropriate plots. Comparisons of Likert scale results at individual time points will be tested using Mann-Whitney U tests. Complications and incidence of re-operations will be assessed using descriptive statistics (counts and percentages). Comparisons between treatment groups for specific complications of interest will be assessed using Chi-squared tests or Fisher's Exact tests. If necessary, missing data will be imputed using multiple imputation techniques.

Economic evaluation

An economic evaluation will be performed from the societal and healthcare perspectives to evaluate the cost-effectiveness of-the bilateral interarcuair decompression compared to a 'classic' laminectomy for patients with INC. When the societal perspective is applied, all costs and consequences relevant to the interventions will be taken into account, whereas only costs accruing to the formal Dutch healthcare sector will be considered when the healthcare perspective is applied. Intervention costs will be estimated using a micro-costing approach. Cost questionnaires will be administered at 3, 6 and 12 weeks covering the entire period and at 6, 12, 18, 24, 36 and 48 months after surgery covering the previous three months. Data will be collected on healthcare utilization, the use of informal care, absenteeism, presenteeism and unpaid productivity losses. Costs will be valued using guideline prices recommended in the Netherlands[46].

For peer review only

For peer review only - http://bmjopen.bmj.com/site/about/guidelines.xhtml

A cost-utility analyses will be conducted with quality adjusted life years (QALYs) as outcome. For estimating QALYs, the patients' EQ-5D-5L health states will be converted into utility scores using the Dutch tariff[43]. Linear interpolation between measurement points will be used to subsequently calculate QALYs. Missing data will be imputed using multiple imputation by chained equations[47]. An incremental cost-utility ratio will be calculated by dividing the difference in costs by the difference in effects. The cost-utility ratio expresses the incremental costs per QALY gained. Bootstrapping techniques will be used to estimate the uncertainty surrounding the cost-effectiveness estimates. Uncertainty will be shown in cost-effectiveness planes and cost-effectiveness acceptability curves, and sensitivity analyses will be performed to test the robustness of the study results.

Data monitoring

This study will be monitored according to a detailed monitoring plan adapted to the risk classification of the Dutch University Federation guidelines. Based on this guideline, the risk classification of this study is regarded negligible. Considerations in this assessment are that this is an investigator-initiated trial, not with vulnerable patients, and while side effects, such as nerve root damage, are known, severe adverse events are extremely rare. Audits may be required by the Medical Ethical Committee or by the regulatory authority inspections and will be granted if necessary. Patients' permission for these audits is obtained with informed consent.

ETHICS AND DISSEMINATION

Research ethics approval

This protocol along with the informed consent forms, recruitment materials and other requested documents will be reviewed and approved by the medical ethical committee of Erasmus MC in Rotterdam with respect to scientific content and compliance with applicable research and human subject regulations.

For peer review only - http://bmjopen.bmj.com/site/about/guidelines.xhtml

Protocol amendments

Any subsequent amendments will be reviewed and approved by the ethical review bodies.

Consent or assent

Surgeons will introduce the trial to patients at the outpatient clinic and will hand them a patient information brochure regarding the main aspects of the trial. Patients will be given five working days to decide if they want to participate in the trial. Trained research nurses will call patients after one week to ask if they wish to partake in the trial. After verbal consent, an appointment will be made with the research nurse, where informed consent forms will be obtained (see appendix 1).

Confidentiality

All study-related information will be stored securely at the study site. All participant information will be stored in locked file cabinets with limited access. All reports, data collection (e.g. CRFS) and administrative forms will be entered into an online data system. All these documents will be identified by a coded study number only to maintain the patient confidentiality. These data will be stored for at least 15 years.

Ancillary and post-trial care

Adverse events (AE) and serious adverse events (SAE) will be monitored. SAE will be reported within 24 hours. The sponsor has an insurance which is in accordance with the legal obligations in the Netherlands. The insurance applies to the damage that becomes apparent during the study or within four years after the end of the study.

Dissemination policy

For peer review only

For peer review only - http://bmjopen.bmj.com/site/about/guidelines.xhtml

427 The final trial results will be communicated to the participants, healthcare professionals, professional organizations and relevant guideline committees in the Netherlands. The results will be published in an international peer-reviewed open-access scientific journal. There are no publication restrictions.

Discussion

In this article, a protocol for a multicentre patient and observer blinded RCT of the SIZE study is presented. Surgical treatment, such as laminectomy or interarcuair decompression, for LSS have been shown to reduce symptoms[10 11]. Due to an abundance of surgical interventions to treat LSS, the surgical management as of now, demonstrates a wide variety of preferred treatments by spine surgeons[48]. To reduce this variety, there is a necessity for randomised controlled trials, to create guidelines on the optimal treatment for LSS[49]. There is an ongoing trial that compares three surgical decompression techniques in patients with lumbar spinal stenosis. Yet, full laminectomy is not applied in this trial, which is still a frequently used surgical technique [48 50]. A recent study compared the radiological and clinical results of bilateral interlaminar decompression and laminectomy[51]. However, this study was a single centre study, was not blinded, and did not include a computerized randomisation. Furthermore, there was no data regarding the cost-effectiveness of both surgical interventions. The direct and indirect costs of an intervention are important determinants, that have to be taken into account by the surgeon, during everyday decision-making. In the SIZE study, we intend to include an economic evaluation to estimate the cost-effectiveness and therefore acquire new information on this topic.

For peer review only - http://bmjopen.bmj.com/site/about/guidelines.xhtml

References

1. Arnoldi CC, Brodsky AE, Cauchoix J, et al. Lumbar spinal stenosis and nerve root entrapment syndromes. Definition and classification. Clin Orthop Relat Res 1976(115):4-5.
2. Ishimoto Y, Yoshimura N, Muraki S, et al. Associations between radiographic lumbar spinal stenosis and clinical symptoms in the general population: the Wakayama Spine Study. Osteoarthritis Cartilage 2013;**21**(6):783-8.
3. Deyo RA, Mirza SK. Trends and variations in the use of spine surgery. Clin Orthop Relat Res 2006;**443**:139-46.
4. Deyo RA, Mirza SK, Martin BI, et al. Trends, major medical complications, and charges associated with surgery for lumbar spinal stenosis in older adults. Jama 2010;**303**(13):1259-65.
5. Amundsen T, Weber H, Lilleas F, et al. Lumbar spinal stenosis. Clinical and radiologic features. Spine (Phila Pa 1976) 1995;**20**(10):1178-86.
6. Evans JG. Neurogenic Intermittent Claudication. Br Med J 1964;**2**(5415):985-7.
7. Fritz JM, Delitto A, Welch WC, et al. Lumbar spinal stenosis: a review of current concepts in evaluation, management, and outcome measurements. Arch Phys Med Rehabil 1998;**79**(6):700-8.
8. Ammendolia C, Stuber KJ, Rok E, et al. Nonoperative treatment for lumbar spinal stenosis with neurogenic claudication. Cochrane Database Syst Rev 2013(8):CD010712.
9. Zaina F, Tomkins-Lane C Fau - Carragee E, Carragee E Fau - Negrini S, et al. Surgical versus non-surgical treatment for lumbar spinal stenosis. (1469-493X (Electronic)).
10. Malmivaara A, Slatis P, Heliovaara M, et al. Surgical or nonoperative treatment for lumbar spinal stenosis? A randomized controlled trial. Spine (Phila Pa 1976) 2007;**32**(1):1-8.
11. Weinstein JN, Tosteson TD, Lurie JD, et al. Surgical versus nonoperative treatment for lumbar spinal stenosis four-year results of the Spine Patient Outcomes Research Trial. Spine (Phila Pa 1976) 2010;**35**(14):1329-38.
12. Gibson JN, Waddell G. Surgery for degenerative lumbar spondylosis. Cochrane Database Syst Rev 2005(4):CD001352.
13. Fox MW, Onofrio BM, Hanssen AD. Clinical outcomes and radiological instability following decompressive lumbar laminectomy for degenerative spinal stenosis: a comparison of patients undergoing concomitant arthrodesis versus decompression alone. J Neurosurg 1996;**85**:793-802.
14. Johnsson KE, Willner S, Johnsson K. Postoperative instability after decompression for lumbar spinal stenosis. Spine 1986;**11**:107-10.
15. Tuite GF, Doran SE, Stern JD, et al. Outcome after laminectomy for lumbar spinal stenosis. Part II: Radiographic changes and clinical correlations. J Neurosurg 1994;**81**:707-15.
16. Thome C, Zevgaridis D, Leheta O, et al. Outcome after less-invasive decompression of lumbar spinal stenosis: a randomized comparison of unilateral laminotomy, bilateral laminotomy, and laminectomy. J Neurosurg Spine 2005;**3**(2):129-41.
17. Mayer HM. *Minimally Invasive Spine Surgery. A Surgical Manual*: Springer, 2005.
18. Oppenheimer JH, DeCastro I, McDonnell DE. Minimally invasive spine technology and minimally invasive spine surgery: a historical review. Neurosurg Focus 2009;**27**(3):E9.
19. Nerland US, Jakola AS, Solheim O, et al. Minimally invasive decompression versus open laminectomy for central stenosis of the lumbar spine: pragmatic comparative effectiveness study. (1756-1833 (Electronic)).
20. Hu SS, Tribus CB, Diab M, et al. Spondylolisthesis and spondylolysis. J Bone Joint Surg Am 2008;**90**(3):656-71.
21. Meyerding HW. Spondylolisthesis. Surg Gynecol Obstet 1932;**54**:371-77.

For peer review only

For peer review only - http://bmjopen.bmj.com/site/about/guidelines.xhtml

22. Aryanpur J, Ducker T. Multilevel lumbar laminotomies: an alternative to laminectomy in the treatment of lumbar stenosis. Neurosurgery 1990;**26**(3):429-32; discussion 33.
23. Hutchinson PJ, Laing RJ, Waran V, et al. Assessing outcome in lumbar disc surgery using patient completed measures. Br J Neurosurg 2000;**14**(3):195-9.
24. Roland M, Morris R. A study of the natural history of low-back pain. Part II: development of guidelines for trials of treatment in primary care. Spine (Phila Pa 1976) 1983;**8**(2):145-50.
25. Roland M, Morris R. A study of the natural history of back pain. Part I: development of a reliable and sensitive measure of disability in low-back pain. Spine (Phila Pa 1976) 1983;**8**(2):141-4.
26. Brouwer S, Kuijer W, Dijkstra PU, et al. Reliability and stability of the Roland Morris Disability Questionnaire: intra class correlation and limits of agreement. Disabil Rehabil 2004;**26**(3):162-5.
27. Smeets R, Koke A, Lin CW, et al. Measures of function in low back pain/disorders: Low Back Pain Rating Scale (LBPRS), Oswestry Disability Index (ODI), Progressive Isoinertial Lifting Evaluation (PILE), Quebec Back Pain Disability Scale (QBPDS), and Roland-Morris Disability Questionnaire (RDQ). Arthritis Care Res (Hoboken) 2011;**63 Suppl 11**:S158-73.
28. Fairbank JC, Pynsent PB. The Oswestry Disability Index. Spine (Phila Pa 1976) 2000;**25**(22):2940-52; discussion 52.
29. van Hooff ML, Spruit M, Fairbank JC, et al. The Oswestry Disability Index (version 2.1a): validation of a Dutch language version. Spine (Phila Pa 1976) 2015;**40**(2):E83-90.
30. Williamson A, Hoggart B. Pain: a review of three commonly used pain rating scales. J Clin Nurs 2005;**14**(7):798-804.
31. Chapman JR, Norvell DC, Hermsmeyer JT, et al. Evaluating common outcomes for measuring treatment success for chronic low back pain. Spine (Phila Pa 1976) 2011;**36**(21 Suppl):S54-68.
32. Bombardier C. Outcome assessments in the evaluation of treatment of spinal disorders: summary and general recommendations. Spine (Phila Pa 1976) 2000;**25**(24):3100-3.
33. Gautschi OP, Corniola MV, Joswig H, et al. The timed up and go test for lumbar degenerative disc disease. J Clin Neurosci 2015;**22**(12):1943-8.
34. Gautschi OP, Smoll NR, Corniola MV, et al. Validity and Reliability of a Measurement of Objective Functional Impairment in Lumbar Degenerative Disc Disease: The Timed Up and Go (TUG) Test. Neurosurgery 2016;**79**(2):270-8.
35. Gautschi OP, Joswig H, Corniola MV, et al. Pre- and postoperative correlation of patient-reported outcome measures with standardized Timed Up and Go (TUG) test results in lumbar degenerative disc disease. Acta Neurochir (Wien) 2016;**158**(10):1875-81.
36. Victor ES, Marc LS. The five-repetition sit-to-stand test: evaluation of a simple and objective tool for the assessment of degenerative pathologies of the lumbar spine. 2018;**29**(4):380.
37. Jones SE, Kon SS, Canavan JL, et al. The five-repetition sit-to-stand test as a functional outcome measure in COPD. Thorax 2013;**68**(11):1015-20.
38. Grelat M, Gouteron A, Casillas JM, et al. Walking Speed as an Alternative Measure of Functional Status in Patients with Lumbar Spinal Stenosis. (1878-8769 (Electronic)).
39. Stienen MN, Gautschi OP, Staartjes VE, et al. Reliability of the 6-minute walking test smartphone application. (1547-5646 (Electronic)).
40. Tosic L, Goldberger E, Maldaner N, et al. Normative data of a smartphone app-based 6-minute walking test, test-retest reliability, and content validity with patient-reported outcome measures. (1547-5646 (Electronic)).
41. Niggemann P, Kuchta J, Grosskurth D, et al. Spondylolysis and isthmic spondylolisthesis: impact of vertebral hypoplasia on the use of the Meyerding classification. The British journal of radiology 2012;**85**(1012):358-62.
42. Herdman M, Gudex C, Lloyd A, et al. Development and preliminary testing of the new five-level version of EQ-5D (EQ-5D-5L). Qual Life Res 2011;**20**(10):1727-36.

For peer review only - http://bmjopen.bmj.com/site/about/guidelines.xhtml

43. M MV, K MV, S MAAE, et al. Dutch Tariff for the Five-Level Version of EQ-5D. Value Health 2016;**19**(4):343-52.
44. Chan AW, Tetzlaff JM, Altman DG, et al. SPIRIT 2013 statement: defining standard protocol items for clinical trials. Ann Intern Med 2013;**158**(3):200-7.
45. Jordan K, Dunn KM, Lewis M, et al. A minimal clinically important difference was derived for the Roland-Morris Disability Questionnaire for low back pain. J Clin Epidemiol 2006;**59**(1):45-52.
46. Versteegh M, Knies S, Brouwer W. From Good to Better: New Dutch Guidelines for Economic Evaluations in Healthcare. Pharmacoeconomics 2016;**34**(11):1071-74.
47. White IR, Royston P, Wood AM. Multiple imputation using chained equations: Issues and guidance for practice. Stat Med 2011;**30**(4):377-99.
48. Overdevest GM, Moojen WA, Arts MP, et al. Management of lumbar spinal stenosis: a survey among Dutch spine surgeons. Acta Neurochir (Wien) 2014;**156**(11):2139-45.
49. Overdevest GM, Jacobs W Fau - Vleggeert-Lankamp C, Vleggeert-Lankamp C Fau - Thomé C, et al. Effectiveness of posterior decompression techniques compared with conventional laminectomy for lumbar stenosis. (1469-493X (Electronic)).
50. Hermansen EA-O, Austevoll IM, Romild UK, et al. Study-protocol for a randomized controlled trial comparing clinical and radiological results after three different posterior decompression techniques for lumbar spinal stenosis: the Spinal Stenosis Trial (SST) (part of the NORDSTEN Study). (1471-2474 (Electronic)).
51. Soliman MAR, Ali A. Decompression of lumbar canal stenosis with a bilateral interlaminar versus classic laminectomy technique: a prospective randomized study. Neurosurg Focus 2019;**46**(5):E3.

AUTHOR'S CONTRIBUTION

J.R.J. Arjun Sharma: JAS

P.S. Gadjradj: PG

Wouter Mooijen: WM

Wilco Peul: WP

Maurits van Tulder: MvT

B.S. Harhangi: BH

JAS contributed to drafting article and took the lead in writing the manuscript. All authors provided critical feedback and helped shape the research and manuscript.

JAS, PG, WM, WP and BH devised the main conceptual ideas and designed the study.

MvT designed the statistical analysis and the economic evaluation.

PG and BH provided funding for conducting this study. JAS, PG, WM, WP, MvT and BH contributed to the study protocol.

For peer review only

For peer review only - http://bmjopen.bmj.com/site/about/guidelines.xhtml

APPENDIX 1

Informed consent materials/form

Interarcuaire decompressie versus laminectomie bij neurogene claudicatie.

- Ik heb de informatiebrief gelezen. Ook kon ik vragen stellen. Mijn vragen zijn voldoende beantwoord. Ik had genoeg tijd om te beslissen of ik meedoe.
- Ik weet dat meedoen vrijwillig is. Ook weet ik dat ik op ieder moment kan beslissen om toch niet mee te doen of te stoppen met het onderzoek. Daarvoor hoef ik geen reden te geven.
- Ik geef toestemming voor het informeren van mijn huisarts/de specialist die mij behandelt dat ik meedoe aan dit onderzoek.
- Ik geef toestemming voor het opvragen van informatie bij de specialist die mij behandelt over de wervelkanaalstenose.
- Ik geef toestemming voor het verzamelen en gebruiken van mijn gegevens voor de beantwoording van de onderzoeksvraag in dit onderzoek.
- Ik weet dat voor de controle van het onderzoek sommige mensen toegang tot al mijn gegevens kunnen krijgen. Die mensen staan vermeld in deze informatiebrief. Ik geef toestemming voor die inzage door deze personen.

<voor zover van toepassing:>

- Ik geef toestemming voor het informeren van mijn huisarts en/of behandelend specialist van onverwachte bevindingen die van belang (kunnen) zijn voor mijn gezondheid.
- Ik geef □ **wel**

  □ **geen**

  toestemming om mijn persoonsgegevens langer te bewaren en te gebruiken voor toekomstig onderzoek op het gebied van mijn aandoening en de onderzochte behandelwijze
- Ik geef □ **wel**

  □ **geen**

  toestemming om mij na dit onderzoek opnieuw te benaderen voor een vervolgonderzoek.
- Ik wil □ **wel**

  □ **niet**

  geïnformeerd worden over welke behandeling ik heb gehad/in welke groep ik zat.

- Ik wil meedoen aan dit onderzoek.

For peer review only

Naam proefpersoon:

Handtekening: Datum : __ / __ / __

Ik verklaar dat ik deze proefpersoon volledig heb geïnformeerd over het genoemde onderzoek.

Als er tijdens het onderzoek informatie bekend wordt die de toestemming van de proefpersoon zou kunnen beïnvloeden, dan breng ik hem/haar daarvan tijdig op de hoogte.

Naam onderzoeker (of diens vertegenwoordiger):

Handtekening: Datum: __ / __ / __

Aanvullende informatie is gegeven door:

Naam:

Functie:

Handtekening: Datum: __ / __ / __

----

* Doorhalen wat niet van toepassing is.

For peer review only

Reporting checklist for protocol of a clinical trial.

Based on the SPIRIT guidelines.

Instructions to authors

Complete this checklist by entering the page numbers from your manuscript where readers will find each of the items listed below.

Your article may not currently address all the items on the checklist. Please modify your text to include the missing information. If you are certain that an item does not apply, please write "n/a" and provide a short explanation.

Upload your completed checklist as an extra file when you submit to a journal.

In your methods section, say that you used the SPIRITreporting guidelines, and cite them as:

Chan A-W, Tetzlaff JM, Altman DG, Laupacis A, Gøtzsche PC, Krleža-Jerić K, Hróbjartsson A, Mann H, Dickersin K, Berlin J, Doré C, Parulekar W, Summerskill W, Groves T, Schulz K, Sox H, Rockhold FW, Rennie D, Moher D. SPIRIT 2013 Statement: Defining standard protocol items for clinical trials. Ann Intern Med. 2013;158(3):200-207

		Reporting Item	Page Number
**Administrative information**			
Title	#1	Descriptive title identifying the study design, population, interventions, and, if applicable, trial acronym	1

For peer review only

Trial registration	#2a	Trial identifier and registry name. If not yet registered, name of intended registry	1
Trial registration: data set	#2b	All items from the World Health Organization Trial Registration Data Set	1
Protocol version	#3	Date and version identifier	1
Funding	#4	Sources and types of financial, material, and other support	1
Roles and responsibilities: contributorship	#5a	Names, affiliations, and roles of protocol contributors	1 and 24
Roles and responsibilities: sponsor contact information	#5b	Name and contact information for the trial sponsor	1
Roles and responsibilities: sponsor and funder	#5c	Role of study sponsor and funders, if any, in study design; collection, management, analysis, and interpretation of data; writing of the report; and the decision to submit the report for publication, including whether they will have ultimate authority over any of these activities	1
Roles and responsibilities: committees	#5d	Composition, roles, and responsibilities of the coordinating centre, steering committee, endpoint adjudication committee, data management team, and	n/a

For peer review only

		other individuals or groups overseeing the trial, if applicable (see Item 21a for data monitoring committee)	
**Introduction**			
Background and rationale	#6a	Description of research question and justification for undertaking the trial, including summary of relevant studies (published and unpublished) examining benefits and harms for each intervention	5
Background and rationale: choice of comparators	#6b	Explanation for choice of comparators	5-6
Objectives	#7	Specific objectives or hypotheses	6
Trial design	#8	Description of trial design including type of trial (eg, parallel group, crossover, factorial, single group), allocation ratio, and framework (eg, superiority, equivalence, non-inferiority, exploratory)	6
**Methods: Participants, interventions, and outcomes**			
Study setting	#9	Description of study settings (eg, community clinic, academic hospital) and list of countries where data will be collected. Reference to where list of study sites can be obtained	7

For peer review only

Eligibility criteria	#10	Inclusion and exclusion criteria for participants. If applicable, eligibility criteria for study centres and individuals who will perform the interventions (eg, surgeons, psychotherapists)	7-8
Interventions: description	#11a	Interventions for each group with sufficient detail to allow replication, including how and when they will be administered	8-9
Interventions: modifications	#11b	Criteria for discontinuing or modifying allocated interventions for a given trial participant (eg, drug dose change in response to harms, participant request, or improving / worsening disease)	n/a
Interventions: adherance	#11c	Strategies to improve adherence to intervention protocols, and any procedures for monitoring adherence (eg, drug tablet return; laboratory tests)	n/a
Interventions: concomitant care	#11d	Relevant concomitant care and interventions that are permitted or prohibited during the trial	9
Outcomes	#12	Primary, secondary, and other outcomes, including the specific measurement variable (eg, systolic blood pressure), analysis metric (eg, change from baseline, final value, time to event), method of aggregation (eg, median, proportion), and time point for each outcome. Explanation of the clinical relevance of chosen efficacy and harm outcomes is strongly recommended	9-14

For peer review only

Participant timeline	#13	Time schedule of enrolment, interventions (including any run-ins and washouts), assessments, and visits for participants. A schematic diagram is highly recommended (see Figure)	15
Sample size	#14	Estimated number of participants needed to achieve study objectives and how it was determined, including clinical and statistical assumptions supporting any sample size calculations	16
Recruitment	#15	Strategies for achieving adequate participant enrolment to reach target sample size	n/a
**Methods: Assignment of interventions (for controlled trials)**			
Allocation: sequence generation	#16a	Method of generating the allocation sequence (eg, computer-generated random numbers), and list of any factors for stratification. To reduce predictability of a random sequence, details of any planned restriction (eg, blocking) should be provided in a separate document that is unavailable to those who enrol participants or assign interventions	16
Allocation concealment mechanism	#16b	Mechanism of implementing the allocation sequence (eg, central telephone; sequentially numbered, opaque,	16

For peer review only

		sealed envelopes), describing any steps to conceal the sequence until interventions are assigned	
Allocation: implementation	#16c	Who will generate the allocation sequence, who will enrol participants, and who will assign participants to interventions	16
Blinding (masking)	#17a	Who will be blinded after assignment to interventions (eg, trial participants, care providers, outcome assessors, data analysts), and how	16
Blinding (masking): emergency unblinding	#17b	If blinded, circumstances under which unblinding is permissible, and procedure for revealing a participant’s allocated intervention during the trial	16
**Methods: Data collection, management, and analysis**			
Data collection plan	#18a	Plans for assessment and collection of outcome, baseline, and other trial data, including any related processes to promote data quality (eg, duplicate measurements, training of assessors) and a description of study instruments (eg, questionnaires, laboratory tests) along with their reliability and validity, if known. Reference to where data collection forms can be found, if not in the protocol	16-17

For peer review only

Data collection plan: retention	#18b	Plans to promote participant retention and complete follow-up, including list of any outcome data to be collected for participants who discontinue or deviate from intervention protocols	17
Data management	#19	Plans for data entry, coding, security, and storage, including any related processes to promote data quality (eg, double data entry; range checks for data values). Reference to where details of data management procedures can be found, if not in the protocol	17
Statistics: outcomes	#20a	Statistical methods for analysing primary and secondary outcomes. Reference to where other details of the statistical analysis plan can be found, if not in the protocol	17-19
Statistics: additional analyses	#20b	Methods for any additional analyses (eg, subgroup and adjusted analyses)	17-19
Statistics: analysis population and missing data	#20c	Definition of analysis population relating to protocol non-adherence (eg, as randomised analysis), and any statistical methods to handle missing data (eg, multiple imputation)	18

Methods: Monitoring

Data monitoring: formal committee	#21a	Composition of data monitoring committee (DMC); summary of its role and reporting structure; statement of whether it is independent from the sponsor and competing interests; and reference to where further	19

For peer review only

		details about its charter can be found, if not in the protocol. Alternatively, an explanation of why a DMC is not needed	
Data monitoring: interim analysis	#21b	Description of any interim analyses and stopping guidelines, including who will have access to these interim results and make the final decision to terminate the trial	19
Harms	#22	Plans for collecting, assessing, reporting, and managing solicited and spontaneously reported adverse events and other unintended effects of trial interventions or trial conduct	20
Auditing	#23	Frequency and procedures for auditing trial conduct, if any, and whether the process will be independent from investigators and the sponsor	n/a
**Ethics and dissemination**			
Research ethics approval	#24	Plans for seeking research ethics committee / institutional review board (REC / IRB) approval	19
Protocol amendments	#25	Plans for communicating important protocol modifications (eg, changes to eligibility criteria, outcomes, analyses) to relevant parties (eg, investigators, REC / IRBs, trial participants, trial registries, journals, regulators)	19

For peer review only

Consent or assent	#26a	Who will obtain informed consent or assent from potential trial participants or authorised surrogates, and how (see Item 32)	20
Consent or assent: ancillary studies	#26b	Additional consent provisions for collection and use of participant data and biological specimens in ancillary studies, if applicable	n/a
Confidentiality	#27	How personal information about potential and enrolled participants will be collected, shared, and maintained in order to protect confidentiality before, during, and after the trial	20
Declaration of interests	#28	Financial and other competing interests for principal investigators for the overall trial and each study site	1 (n/a)
Data access	#29	Statement of who will have access to the final trial dataset, and disclosure of contractual agreements that limit such access for investigators	20
Ancillary and post trial care	#30	Provisions, if any, for ancillary and post-trial care, and for compensation to those who suffer harm from trial participation	20
Dissemination policy: trial results	#31a	Plans for investigators and sponsor to communicate trial results to participants, healthcare professionals, the public, and other relevant groups (eg, via publication, reporting in results databases, or other data sharing arrangements), including any publication restrictions	20

For peer review only

Dissemination policy: authorship	#31b	Authorship eligibility guidelines and any intended use of professional writers	n/a
Dissemination policy: reproducible research	#31c	Plans, if any, for granting public access to the full protocol, participant-level dataset, and statistical code	n/a

Appendices

Informed consent materials	#32	Model consent form and other related documentation given to participants and authorised surrogates	25-26
Biological specimens	#33	Plans for collection, laboratory evaluation, and storage of biological specimens for genetic or molecular analysis in the current trial and for future use in ancillary studies, if applicable	n/a

The SPIRIT checklist is distributed under the terms of the Creative Commons Attribution License CC-BY-ND 3.0. This checklist was completed on 05. January 2020 using https://www.goodreports.org/, a tool made by the EQUATOR Network in collaboration with Penelope.ai

For peer review only

BMJ Open

SIZE study: study protocol of a multi-centre, randomised controlled trial to compare the effectiveness of an interarcuair decompression versus extended decompression in patients with intermittent neurogenic claudication caused by lumbar spinal stenosis.

Journal:	*BMJ Open*
Manuscript ID	bmjopen-2020-036818.R2
Article Type:	Protocol
Date Submitted by the Author:	26-Aug-2020
Complete List of Authors:	Arjun Sharma, Jamie; Erasmus Medical Center, Neurosurgery<br>Gadjradj, Pravesh; Erasmusmc, Neurosurgery<br>Peul, Wilco; Leiden University Medical Center, Neurosurgery<br>van Tulder, Maurits; University of Amsterdam, Health Sciences<br>Moojen, Wouter; Medical Centre Haaglanden, Neurosurgery; Leiden University Medical Center, Neurosurgery<br>Harhangi, Biswadjiet; Erasmus MC, ; Erasmus Medical Center, Neurosurgery
**Primary Subject Heading**:	Surgery
Secondary Subject Heading:	Surgery
Keywords:	NEUROSURGERY, Spine < ORTHOPAEDIC & TRAUMA SURGERY, Neurosurgery < SURGERY

SCHOLARONE™
Manuscripts

I, the Submitting Author has the right to grant and does grant on behalf of all authors of the Work (as defined in the below author licence), an exclusive licence and/or a non-exclusive licence for contributions from authors who are: i) UK Crown employees; ii) where BMJ has agreed a CC-BY licence shall apply, and/or iii) in accordance with the terms applicable for US Federal Government officers or employees acting as part of their official duties; on a worldwide, perpetual, irrevocable, royalty-free basis to BMJ Publishing Group Ltd ("BMJ") its licensees and where the relevant Journal is co-owned by BMJ to the co-owners of the Journal, to publish the Work in this journal and any other BMJ products and to exploit all rights, as set out in our licence.

The Submitting Author accepts and understands that any supply made under these terms is made by BMJ to the Submitting Author unless you are acting as an employee on behalf of your employer or a postgraduate student of an affiliated institution which is paying any applicable article publishing charge ("APC") for Open Access articles. Where the Submitting Author wishes to make the Work available on an Open Access basis (and intends to pay the relevant APC), the terms of reuse of such Open Access shall be governed by a Creative Commons licence – details of these licences and which Creative Commons licence will apply to this Work are set out in our licence referred to above.

Other than as permitted in any relevant BMJ Author's Self Archiving Policies, I confirm this Work has not been accepted for publication elsewhere, is not being considered for publication elsewhere and does not duplicate material already published. I confirm all authors consent to publication of this Work and authorise the granting of this licence.

For peer review only

SIZE study: study protocol of a multi-centre, randomised controlled trial to compare the effectiveness of an interarcuair decompression versus extended decompression in patients with intermittent neurogenic claudication caused by lumbar spinal stenosis.

Article type: design article

J.R.J. Arjun Sharma, BSc[1] , P.S. Gadjradj MD[2], W.C. Peul MD, PhD[2], MBA, M.W. van Tulder PhD[3], W.A. Moojen MD, PhD, MSc[2] , B.S. Harhangi MD, PhD, MSc[1] for the SIZE-study group

1. Department of Neurosurgery, Erasmus MC, University Medical Centre, Rotterdam, The Netherlands
2. Department of Neurosurgery, Haaglanden Medical Centre & Leiden University Medical Centre and Haga Teaching Hospital, The Netherlands
3. Department of Health Sciences, Faculty of Science, Vrije Universiteit Amsterdam, MOVE research institute Amsterdam, Amsterdam, the Netherlands.

Protocol version: 3
Funding sources/sponsor: This work was supported by MRace grant, number 2017-17106 (Institutional Grant of Erasmus Medical Centre)
IRB approval status: MEC- 2018-093
Trial registration/ClinicalTrials.gov: NCT03480893
Conflicts of Interest: None declared.

Abstract word count: 269
Manuscript word count: 4083 [excluding references, figures, and tables]
References: 51
Figures: 0
Tables: 1
Supplementary material: 0
Keywords: Spinal Stenosis/surgery, Decompression, Lumbar Vertebrae/surgery, Humans, Laminectomy
Running title: The SIZE study.

Acknowledgements: the SIZE-study group consists, except for the authors, out of
Drs. H.P. van Putten
Dr. R. van Rolde

The authors would furthermore gratefully acknowledge Katya Mauff for statistical advice.

#Corresponding author:
B.S. Harhangi MD, PhD, MSc
Department of Neurosurgery
Erasmus Medical Centre, Rotterdam, the Netherlands
Dr. Molewaterplein 40, 3015 GD
Postbus 2040, 3000 CA Rotterdam
b.s.harhangi@erasmusmc.nl

For peer review only - http://bmjopen.bmj.com/site/about/guidelines.xhtml

Abbreviations:

LSS: Lumbar Spinal Stenosis

INC: Intermittent Neurogenic Claudication

MRI: Magnetic Resonance Imaging

ODI: Oswestry Disability Index

NRS: Numeric Rating Scale

RMDQ: Roland Morris Disability Questionnaire

TUG: Timed-Up and Go Test

6MWT: 6-Minute Walking Test

For peer review only

For peer review only - http://bmjopen.bmj.com/site/about/guidelines.xhtml

ABSTRACT

Introduction

Intermittent neurogenic claudication (INC) is often caused by lumbar spinal stenosis (LSS). Laminectomy is considered a frequently used surgical technique for LSS. Previous studies have shown that laminectomy can potentially cause lumbar instability. Less invasive techniques, preserving midline structures including the bilateral small size interarcuair decompression, are currently applied. Due to lack of evidence and consensus, surgeons have to rely on their training and own experiences to choose the best surgical techniques for their patients. Hence an observer and patient blinded multi-centre, randomised controlled trial was designed to determine the effectiveness and cost-effectiveness of bilateral interarcuair decompression versus laminectomy for LSS.

Methods and analysis

174 patients above 40 years with at least 12 weeks of complaints of INC will be recruited. Patients are eligible for inclusion if they have a clinical indication for surgery for INC with a MRI showing signs of LSS. Patients will be randomised to laminectomy or bilateral interarcuair decompression. The primary outcome is functional status measured with the Roland Morris Disability Questionnaire at 12 months. Secondary outcomes consist of pain intensity, self-perceived recovery, functional status measured with the Oswestry Disability Index, and a physical examination. Outcome measurement moments will be scheduled at 3 and 6 weeks, and at 3, 6, 12, 18, 24, 36 and 48 months after surgery. Physical examination will be performed at 6 weeks, and 12, 24 and 48 months. An economic evaluation will be performed and questionnaires will be used to collect cost data.

Ethics and dissemination

The Medical Ethical Committee of the Erasmus Medical Centre Rotterdam approved this study (NL.65826.078.18). The results will be published in an international peer-reviewed journal.

Trial registration: ClinicalTrials.gov Identifier: NCT03480893

For peer review only

For peer review only - http://bmjopen.bmj.com/site/about/guidelines.xhtml

ARTICLE SUMMARY

'Strengths and limitations of this study'

- Patient and observer blinded randomised controlled design.
- Both patient-reported outcomes and objective functional test outcomes will be measured.
- Inclusion of an economic evaluation.
- Instability as outcome may not be measurable in two years of follow-up.
- Diversity in terminology on posterior decompression techniques may lead to variability on both surgical techniques.

For peer review only

For peer review only - http://bmjopen.bmj.com/site/about/guidelines.xhtml

INTRODUCTION

Lumbar spinal stenosis (LSS) is described as the narrowing of the diameter of the spinal and nerve root canals, which can cause compression of the neural structures in the canals[1]. LSS is a common medical condition in the aging population and is considered the most common reason for lumbar spine surgery for people over the age of 65[2-4]. Intermittent neurogenic claudication (INC) is the most important symptom of LSS, with complaints of leg pain and heaviness of the legs, usually bilateral, which exacerbate during standing and walking. Apart from leg pain, back pain is often also present[5]. As the symptoms aggravate, the ability to walk can become considerably limited, which is the dominant functional impairment and the most common reason for seeking medical care[6 7].

Conservative treatment, such as physical therapy and pain medication, is the first treatment option and may give some relief of symptoms[8]. Previous research shows that there is sparse evidence of the effectiveness of surgery compared to non-operative treatment[9]. However, usually when conservative treatment fails, surgical treatment is considered the next option[10 11]. The first technique described to widen the lumbar spinal canal is the laminectomy technique, also known as wide bony decompression , which is a widely used surgical technique for patients with INC caused by LSS[12]. Given that patients often experience post-operative back pain after laminectomy, it is hypothesized that laminectomy is a ground for potential lumbar instability and iatrogenic scoliosis[13-15]. Performing a procedure with potentially more complications in an elderly population could be considered as doubtful. Hence, less invasive techniques, such as the bilateral small size interarcuair decompression, otherwise known as a bilateral laminotomy, limited bony decompression or interlaminar decompression, were developed and implemented in clinical practice. These techniques require less bone tissue to be removed, which possibly can reduce the prevalence of lumbar instability in these patients and consequently improve functioning[16]. Furthermore, it is believed that, compared to laminectomy, these less invasive techniques

For peer review only - http://bmjopen.bmj.com/site/about/guidelines.xhtml

have the ability to decompress the nerves while preserving the spinal integrity. However, the risk of an insufficient decompression may be higher, possibly resulting in reoperations due to residual stenosis[16-18]. The presumption is often made that, after a wide decompression, recurrence of the complaints are scarce. The opinions on this matter are diverse and subject to debate.[19]. Therefore, a randomised controlled trial is needed to clarify whether bilateral interarcuair decompression is effective and cost-effective compared to a classical laminectomy.

Study objectives

The primary objective of this study is to determine whether bilateral interarcuair decompression is more effective than laminectomy in patients with INC caused by LSS. The secondary objective is to evaluate if bilateral interarcuair decompression is more cost-effective than laminectomy.

Study design

The study is designed as a multi-centre, patient and observer blinded randomised controlled trial with an economic evaluation alongside. Participants will be allocated to one of the two groups: laminectomy or interarcuair decompression.

For peer review only - http://bmjopen.bmj.com/site/about/guidelines.xhtml

METHODS

Study setting/population

In total 174 patients with a MRI-confirmed LSS will be recruited. All patients who will be included should have an indication for surgery due to LSS with minimally 12 weeks of complaints of INC, as is customary in clinical practice, and failed conservative treatment. Patients will be recruited from two hospitals and one private health clinic in the Netherlands:

- Erasmus Medical Centre in Rotterdam.
- Maasstad Hospital in Rotterdam.
- Park Medical Centre in Rotterdam.

Eligibility criteria

In order to be suitable to participate in this study, patients must meet all of the following criteria:

Inclusion criteria

- At least 12 weeks of complaints of INC with a leg pain level above 3 on a numeric rating scale (NRS), without sufficient response to conservative management.
- Indication for an operation according to consensus of surgeon
- MRI demonstrating LSS
- Age above 40 years
- Sufficient knowledge of the Dutch language in order to comprehend the questionnaires and patient information
- Written informed consent

Exclusion criteria

For peer review only - http://bmjopen.bmj.com/site/about/guidelines.xhtml

149 Patients who meet any of the following criteria will be excluded from participating in this study.

- History of lumbar spine surgery
- More than 2 lumbar levels needing surgery
- Degenerative spondylolisthesis greater than Meyerding grade I (on a scale of I to V)[20 21]
- Concomitant scoliosis or disc herniation
- Severe comorbid medical disorder (American Society of Anesthesiologists > 3)
- Serious psychopathologic disorder
- Pregnancy
- Active malignancy
- Plans to move abroad during study period.

Eligible patients will be randomised in a 1:1 ratio to the two groups.

Interventions

Intervention: bilateral small size interarcuair decompression

The intervention consists of the interarcuair decompression, otherwise known as a bilateral laminotomy, limited bony decompression or an interlaminar decompression. General anaesthesia is to be administered. A midline skin incision is made, after which the paravertebral muscles are dissected subperiosteally and retracted bilaterally. Decompression will be applied through decompression of the ligamentum flavum and partial laminotomy if necessary. The lateral recess will be opened bilaterally and a partial medial facetectomy will be performed to decompress neuronal structures including the exciting nerve root. The lamina will be removed up to the ligamentum flavum. Interspinous ligaments are spared.[22] The wound is closed in layers. Patients are operated with a loupe magnification or microscope depending on the surgeon's preference.

For peer review only - http://bmjopen.bmj.com/site/about/guidelines.xhtml

Control group: conventional laminectomy

The control group consists of conventional laminectomy, otherwise known as wide bony decompression. General anaesthesia is to be administered. A midline skin incision is made over the spinous processes. The laminae of the affected level(s) are exposed subperiosteally, and the supraspinous ligament is incised. The spinous process is removed. The supra and interspinous ligament of the affected level is removed by drill or Kerrison punched. Both laminae are removed at the affected level, leaving the facet joint intact. The lateral recess is opened bilaterally and a partial medial facetectomy will be performed to decompress neuronal structures including the exciting nerve root.[16]. To clarify, when a single level stenosis is present (e.g. L4-L5), both laminae L4 and L5 will be removed. When a double level stenosis (e.g. L3-L4 and L4-L5) is present, three laminae (L3, L4 and L5) will be removed. The wound is closed in layers with or without a suction drain. Patients will be operated with loupe magnification or microscope depending on the surgeon's preference.

Use of co-intervention

Pain medication will be provided to patients after surgery, should this be necessary. Further, the use of co-interventions will be tracked by cost questionnaires, in which medication usage and any health care utilization is monitored throughout the follow-up period.

Outcomes

Primary outcomes

The primary outcome is a functional status measured using the Roland-Morris Disability Questionnaire (RMDQ). This is a 24-point questionnaire that is designed to assess the functional status in patients with low back pain. This outcome measure has been identified as one of the most commonly used outcomes in a population with chronic low back pain[23-27].

For peer review only

For peer review only - http://bmjopen.bmj.com/site/about/guidelines.xhtml

194 ***Secondary outcomes***

195 *Oswestry Disability Index (ODI)*

196 The ODI is one of the principal condition-specific outcome measures used in de management of spinal disorders[28]. The current version 2.1a will be used[29]. It consists of 10 questions, each with six possible answers and each answer option receives a score of 0 to 5 points, yielding a score range between 0 and 50, which is scaled to a 100% range. The questions focus on a range of daily physical functions and how the back or leg pain is affecting the patient's ability to manage in everyday life.

201 *Numeric Rating Scale for leg and low back pain*

202 This parameter will measure the experienced pain intensity in both legs (affected and non-affected) and back. Pain intensity will be rated on an 11-point scale, varying from 0 representing 'no pain' to 10 'the most terrible pain imaginable'. Patients are not permitted to see the pain score indicated during previous visits. Reliability and validity of NRS have been shown[30].

206 *Short Form 36 (SF-36)*

207 The quality of life will be measured with the Dutch version of the Short Form 36 (SF36). The SF36 has been applied and successfully validated in populations with low back pain[31]. This questionnaire relates to the analysis of the general functional status of the patient. The questionnaire is divided into eight health concepts: (1) physical functioning, (2) role limitations because of physical health problems, (3) bodily pain, (4) social functioning, (5) general mental health (psychological distress and well-being); (6) emotional role limitations; (7) vitality and (8) general health perceptions. The scores of the different health concepts are added up into a scale of 0-100. A higher score reflects a better general health condition. The physical and mental component summary will be assessed separately.

For peer review only - http://bmjopen.bmj.com/site/about/guidelines.xhtml

Patients' perceived recovery

Recovery will be measured using a seven-point Likert scale. The score on this scale varies from 'complete recovery' to 'worse than ever'. The outcomes 'complete recovery and 'almost complete recovery' will be dichotomized and considered as recovered[32].

Patients' satisfaction

Patients' satisfaction of change and satisfaction of treatment will be assessed using a seven-point Likert scale, ranging from 'completely satisfied' to 'completely dissatisfied'. 'Completely satisfied' and 'almost completely satisfied' will be defined as good outcome.

Physical examination

A physical examination will be performed before surgery, at 6 weeks, 12, 24 and 48 months after surgery. This examination will include a brief neurologic examination, the Timed Up and Go Test (TUG), the Timed Chair-Stand Test, the Six-Minute Walking Test (6MWT) and patient's weight.

a. *Neurologic examination*

   The neurologic examination will include the muscle strength of the quadriceps, the iliopsoas, the hamstrings, the gastrocnemicus and the tibialis anterior muscle, which will be noted as a difference in strength between both legs. We will evaluate if there is a difference in muscle strength between the affected leg and the other leg.

b. *Timed Up and Go Test (TUG)*

   Patients are asked to sit in a chair with their arms resting on the armrests. On direction of the research nurse, patients have to walk as fast as possible (without running) to a wall at 3-m distance of the chair. When they have reached that wall, a turn of 180 degrees is made and they walk directly back to the chair and sit down. The research nurse will measure the time (in seconds) it takes for a patient, to perform this action. The time is stopped when the patients

For peer review only

For peer review only - http://bmjopen.bmj.com/site/about/guidelines.xhtml

are seated again[33]. Patients need to perform this test, three times. Patients are allowed to wear their regular shoes and use a walking aid, if necessary. The TUG test has been described as a valid, reliable tool in the assessment of objective functional impairment in patients with lumbar degenerative disc disease[34 35]. However, this has not been done for LSS. The validation for LSS will be conducted in this study.

c. *Timed Chair-Stand Test*

Patients are asked to sit down on an armless chair, firmly placed against a wall, with their arms folded across their chest. They are instructed to place their feet flat on the ground. To become familiarized with the exercise, the patients are asked to stand up and sit down again without using their arms. If assistance is required during this action, or this movement cannot be completed by the patient, the test is abandoned. If the manoeuvre is possible, the patient will be asked to, on the word "go", stand up entirely and sit down again, 5 times as quick as possible. The research nurse will time the 5 movements in seconds from the command "go" to the completed fifth stand. The measured time will be noted as the score. If the patient is unable to perform the test 5 times, a maximum score will be noted of 30 seconds[36 37].

d. *Six-Minute Walking Test (6MWT)*

This test is performed to assess the gait pattern, walking speed and physical endurance of patients. The patient will be requested to walk at such a speed that at the end of 6 minutes, the patient will have the feeling of maximum output[38]. Before taking the test, the patient will be given the instructions to install the 6MWT application on their cell phone[39 40]. During these 6 minutes this application will measure the walking distance. During the walking test, the patient is permitted to use a walking aid and/or orthosis which will be noted.

For peer review only - http://bmjopen.bmj.com/site/about/guidelines.xhtml

Functional lumbar X-rays

Functional X-rays will be obtained from all patients. The X-ray will be made standing from AP and lateral position to assess spondylolisthesis. X-rays will also be made in maximum flexion and extension position. Degenerative spondylolisthesis is defined as a vertebral slip of at least 3 mm[41]. After the measurement of the vertebral slip, this will be classified according to the Meyerding classification. This will be obtained at 6 weeks postoperatively, for level verification and for verification of correct procedure and after two years postoperatively to look for possible instability.

EuroQol-5-dimension- 5-levels

The EuroQol (EQ-5D-5L) is a validated instrument to assess health-related quality of life (QOL). The tool measures five dimensions:

- Mobility
- Self-care
- Pain/discomfort
- Daily activities
- Anxiety

Each dimension consists of one item, in which five levels are distinguished (no problems, minor problems, moderate problems, major problems, severe problems, unable to). The Dutch tariff of the EQ-5D-5L will be used to calculate the quality-adjusted life years (QALYs)[42 43].

Cost-diaries

Based on a diary with made costs, which will be completed by patients, insight will be obtained in the need for care, working participation and direct- and indirect medical cost. Direct medical cost will be estimated on basis of the cost centre method. In addition it is expected that the study population will consist of elderly people who may be retired. For the cost calculation additional costs, such as help in house, transport, help from family etc. will be taken in account.

For peer review only - http://bmjopen.bmj.com/site/about/guidelines.xhtml

For an estimation of direct- and indirect cost permission of the patient will be asked to request the total amount of cost incurred during the research period from the insurance company. No consideration by participation in the investigation as compared to daily practice will be deducted. The additional investigational costs include mainly the additional visits to the outpatient clinic.

The patient will be requested to keep a diary for the financial aspects of the consequences of INC and corresponding treatment. The patient will be requested to record the following items:

- Visits to GP
- Visits to physiotherapist
- Visits to specialist
- Alternative medicines and devices (e.g. rollator, etc.)
- Number of days of hospitalization
- Pain medication; dosage and frequency
- Illness-related days of absence at work, if any
- Cost of loss production and substitute manpower, if any
- Additional travelling expenses on account of INC
- Help in house holding

Complication and re-operation incidence

Immediately after surgery, the surgeon will report any perioperative complications such as cerebrospinal fluid leakage, nerve root damage and if the operation was implemented at the wrong vertebral level. Additionally, a systematic assessment of complications (including urinary tract infection, progressive neurological deficit and secondary bleeding) will be carried out by the surgeon and the research nurse shortly after discharge.

Reoperation is considered a bad outcome. The incidence of reoperation as well as perioperative morbidity will be assessed in both groups, using surgical parameters such as blood loss, time of surgery and length of hospital stay.

We used the SPIRIT checklist when writing our report[44].

For peer review only - http://bmjopen.bmj.com/site/about/guidelines.xhtml

For peer review only

314 **Patient and public involvement**

315 No patients involved.

316

For peer review only - http://bmjopen.bmj.com/site/about/guidelines.xhtml

Table 1: Participant timeline schedule

Table	Visit plan and case report forms											
	Intake	Surgery (t_0)	1-2 day(s) following surgery	FU 3 weeks	FU 6 weeks	FU 3 months	FU 6 months	FU 12 months	FU 18 months	FU 24 months	FU 36 months	FU 48 months
Visit surgeon	x				x							
Research nurse visit	x				x			x		x		x
Randomisation		X										
Surgery		X										
Discharge			x									
Physical examination	x				x			x		x		x
Functional x-rays	x				x					x		
Roland Morris Questionnaire	x			x	x	x	x	x		x	x	x
Oswestry Disability Index (ODI)	x			x	x	x	x	x		x	x	x
NRS leg and back pain	x			x	x	x	x	x		x	x	x
Patient self-received recovery and satisfaction	x			x	x	x	x	x		x	x	x
EuroQol-5D	x			x	x	x	x	x		x	x	x
Cost questionnaires	x			x	x	x	x	x	x	x		
Short Form 36 (SF-36)	x			x	x	x	x	x		x	x	x
Revisit and complications	WITH OCCURRENCE											

For peer review only

For peer review only - http://bmjopen.bmj.com/site/about/guidelines.xhtml

Sample Size

The sample size of this study is calculated for the primary endpoint of group difference in the RMDQ at 12 months. According to literature, results obtained after interarcuair decompression should be 3 points lower on the RMDQ compared to those for the laminectomy group in order to detect superiority[45].

Hence the sample size of this study is based on a superiority design, using a delta of 3, an assumed true mean difference of 0 and a pooled standard deviation of 6. Using an alpha of 0.05, and a power of 90%, we calculated a sample size of 69 patients per group. Accounting for a 20% loss in follow-up, we will therefore enrol 174 patients (87 patients per group).

Allocation

Computerized block randomisation will take place after the patient has been anaesthetised. Allocation concealment will be assured by using ALEA, a web-based data system supervised by the Clinical Trial Centre (CTC) of the Erasmus MC. Variable block sizes of 4, 6 and 8 will be used and stratified by treatment centre. Patients will be randomised in a 1:1 ratio to laminectomy or bilateral interarcuair decompression. Each patient will be given a unique study number.

Blinding

All researchers and patients will be blinded for the treatment. This is possible, because there are no fundamental differences between both procedures (e.g. general anaesthesia, spinal incision, inpatient procedure). The statistical analyses will be performed blinded. The surgeon cannot be blinded.

For peer review only - http://bmjopen.bmj.com/site/about/guidelines.xhtml

For peer review only

Data collection methods

All patient reported outcomes will be collected preoperatively and after 3 weeks, 6 weeks, 3 months, 6 months, 12 months, 18 months, 24 months, 36 months and 48 months (see Table 1). Patients will receive an email with a link, reminding them of the upcoming data collection and requesting them to fill in the web-based questionnaires. If the questionnaires are not completed, the patient will receive a reminder after one and two weeks. After two weeks, non-responders will be contacted by phone.

Data management

Data from initial visits to the hospital, follow-up visits and questionnaires will be entered into a database via an electronic data system (Gemstracker). This data will be noted and analysed by using coded information (study numbers) without any personal identifiers. Data will be stored via the secure data management system of the trial coordination centre of the Erasmus MC.

Statistical analysis

Patient characteristics will be summarized per treatment group to determine whether the randomisation was balanced. Continuous variables will be presented using means and standard deviations, or in the case of non-normally distributed variables using medians and interquartile ranges. Normality will be graphically assessed. Categorical variables will be presented using counts and percentages. The primary analysis will study differences in the primary outcome measure (RMDQ). The primary endpoint of interest is 12 months after randomisation. The results of the RMDQ will be assessed using generalized linear mixed models (GLMM), to account for the repeated measurements within patients and the multi-centre stratification.

The secondary outcomes (ODI, NRS leg and back pain score, TUG test, TCST and the SF-36) will be similarly assessed using GLMMs and will be analysed for exploratory purposes. In all analyses the

For peer review only

primary assessment of treatment effect will be the estimate of the main effect within the appropriate model at 12 months, adjusted for the stratification factors and main covariates. Time to recovery will be analysed using survival analysis (Cox proportional hazards). At 24, 36 and 48 months, the GLMM repeated-measurement analyses using the compound symmetry covariance structure will be used while *group* (interarcuair and conventional), *time* (moments of measurement) and the *interaction* between Group and Time (Group x Time) will be entered as independent variables. Dependent variables are the primary and secondary outcome parameters. Likert scales for self-reported patient recovery and satisfaction will be assessed using descriptive statistics (counts and percentages) and appropriate plots. Comparisons of Likert scale results at individual time points will be tested using Mann-Whitney U tests. Complications and incidence of re-operations will be assessed using descriptive statistics (counts and percentages). Comparisons between treatment groups for specific complications of interest will be assessed using Chi-squared tests or Fisher's Exact tests. If necessary, missing data will be imputed using multiple imputation techniques.

Economic evaluation

An economic evaluation will be performed from the societal and healthcare perspectives to evaluate the cost-effectiveness of-the bilateral interarcuair decompression compared to a 'classic' laminectomy for patients with INC. When the societal perspective is applied, all costs and consequences relevant to the interventions will be taken into account, whereas only costs accruing to the formal Dutch healthcare sector will be considered when the healthcare perspective is applied. Intervention costs will be estimated using a micro-costing approach. Cost questionnaires will be administered at 3, 6 and 12 weeks covering the entire period and at 6, 12, 18, 24, 36 and 48 months after surgery covering the previous three months. Data will be collected on healthcare utilization, the use of informal care, absenteeism, presenteeism and unpaid productivity losses. Costs will be valued using guideline prices recommended in the Netherlands[46].

For peer review only

388 A cost-utility analyses will be conducted with quality adjusted life years (QALYs) as outcome. For estimating QALYs, the patients' EQ-5D-5L health states will be converted into utility scores using the Dutch tariff[43]. Linear interpolation between measurement points will be used to subsequently calculate QALYs. Missing data will be imputed using multiple imputation by chained equations[47]. An incremental cost-utility ratio will be calculated by dividing the difference in costs by the difference in effects. The cost-utility ratio expresses the incremental costs per QALY gained. Bootstrapping techniques will be used to estimate the uncertainty surrounding the cost-effectiveness estimates. Uncertainty will be shown in cost-effectiveness planes and cost-effectiveness acceptability curves, and sensitivity analyses will be performed to test the robustness of the study results.

Data monitoring

This study will be monitored according to a detailed monitoring plan adapted to the risk classification of the Dutch University Federation guidelines. Based on this guideline, the risk classification of this study is regarded negligible. Considerations in this assessment are that this is an investigator-initiated trial, not with vulnerable patients, and while side effects, such as nerve root damage, are known, severe adverse events are extremely rare. Audits may be required by the Medical Ethical Committee or by the regulatory authority inspections and will be granted if necessary. Patients' permission for these audits is obtained with informed consent.

ETHICS AND DISSEMINATION

Research ethics approval

This protocol along with the informed consent forms, recruitment materials and other requested documents were reviewed and approved by the medical ethical committee of Erasmus MC in Rotterdam with respect to scientific content and compliance with applicable research and human subject regulations.

For peer review only - http://bmjopen.bmj.com/site/about/guidelines.xhtml

411 **Protocol amendments**

412 Any subsequent amendments will be reviewed and approved by the ethical review bodies.

413 **Consent or assent**

414 Surgeons will introduce the trial to patients at the outpatient clinic and will hand them a patient
415 information brochure regarding the main aspects of the trial. Patients will be given five working days to
416 decide if they want to participate in the trial. Trained research nurses will call patients after one week to
417 ask if they wish to partake in the trial. After verbal consent, an appointment will be made with the
418 research nurse, where informed consent forms will be obtained (see appendix 1).

419 **Confidentiality**

420 All study-related information will be stored securely at the study site. All participant information will be
421 stored in locked file cabinets with limited access. All reports, data collection (e.g. CRFs) and
422 administrative forms will be entered into an online data system. All these documents will be identified
423 by a coded study number only to maintain the patient confidentiality. These data will be stored for at
424 least 15 years.

425 **Ancillary and post-trial care**

426 Adverse events (AE) and serious adverse events (SAE) will be monitored. SAE will be reported within 24
427 hours. The sponsor has an insurance which is in accordance with the legal obligations in the
428 Netherlands. The insurance applies to the damage that becomes apparent during the study or within
429 four years after the end of the study.

430

431

432

For peer review only

433 **Dissemination policy**

434 The final trial results will be communicated to the participants, healthcare professionals, professional
435 organizations and relevant guideline committees in the Netherlands. The results will be published in an
436 international peer-reviewed open-access scientific journal. There are no publication restrictions.

437 **Data sharing plan**

438 When primary analyses are conducted and the final report has been published, an anonymized data set
439 will be available upon reasonable request.

440 **Discussion**

441 In this article, a protocol for a multicentre patient and observer blinded RCT of the SIZE study is
442 presented. Surgical treatment, such as laminectomy or interarcuair decompression, for LSS have been
443 shown to reduce symptoms[10 11]. Due to an abundance of surgical interventions to treat LSS, the surgical
444 management as of now, demonstrates a wide variety of preferred treatments by spine surgeons[48]. To
445 reduce this variety, there is a necessity for randomised controlled trials, to create guidelines on the
446 optimal treatment for LSS[49]. There is an ongoing trial that compares three surgical decompression
447 techniques in patients with lumbar spinal stenosis. Yet, conventional laminectomy is not applied in this
448 trial, which is still a frequently used surgical technique [48 50]. A recent study compared the radiological
449 and clinical results of bilateral interlaminar decompression and laminectomy[51]. However, this study was
450 a single centre study, was not blinded, and did not include a computerized randomisation. Furthermore,
451 there was no data regarding the cost-effectiveness of both surgical interventions. The direct and indirect
452 costs of an intervention are important determinants, that have to be taken into account by the surgeon,
453 during every day decision-making. In the SIZE study, we intend to include an economic evaluation to
454 estimate the cost-effectiveness and therefore acquire new information on this topic.

455

For peer review only - http://bmjopen.bmj.com/site/about/guidelines.xhtml

References

1. Arnoldi CC, Brodsky AE, Cauchoix J, et al. Lumbar spinal stenosis and nerve root entrapment syndromes. Definition and classification. Clin Orthop Relat Res 1976(115):4-5.
2. Ishimoto Y, Yoshimura N, Muraki S, et al. Associations between radiographic lumbar spinal stenosis and clinical symptoms in the general population: the Wakayama Spine Study. Osteoarthritis Cartilage 2013;**21**(6):783-8.
3. Deyo RA, Mirza SK. Trends and variations in the use of spine surgery. Clin Orthop Relat Res 2006;**443**:139-46.
4. Deyo RA, Mirza SK, Martin BI, et al. Trends, major medical complications, and charges associated with surgery for lumbar spinal stenosis in older adults. Jama 2010;**303**(13):1259-65.
5. Amundsen T, Weber H, Lilleas F, et al. Lumbar spinal stenosis. Clinical and radiologic features. Spine (Phila Pa 1976) 1995;**20**(10):1178-86.
6. Evans JG. Neurogenic Intermittent Claudication. Br Med J 1964;**2**(5415):985-7.
7. Fritz JM, Delitto A, Welch WC, et al. Lumbar spinal stenosis: a review of current concepts in evaluation, management, and outcome measurements. Arch Phys Med Rehabil 1998;**79**(6):700-8.
8. Ammendolia C, Stuber KJ, Rok E, et al. Nonoperative treatment for lumbar spinal stenosis with neurogenic claudication. Cochrane Database Syst Rev 2013(8):CD010712.
9. Zaina F, Tomkins-Lane C Fau - Carragee E, Carragee E Fau - Negrini S, et al. Surgical versus non-surgical treatment for lumbar spinal stenosis. (1469-493X (Electronic)).
10. Malmivaara A, Slatis P, Heliovaara M, et al. Surgical or nonoperative treatment for lumbar spinal stenosis? A randomized controlled trial. Spine (Phila Pa 1976) 2007;**32**(1):1-8.
11. Weinstein JN, Tosteson TD, Lurie JD, et al. Surgical versus nonoperative treatment for lumbar spinal stenosis four-year results of the Spine Patient Outcomes Research Trial. Spine (Phila Pa 1976) 2010;**35**(14):1329-38.
12. Gibson JN, Waddell G. Surgery for degenerative lumbar spondylosis. Cochrane Database Syst Rev 2005(4):CD001352.
13. Fox MW, Onofrio BM, Hanssen AD. Clinical outcomes and radiological instability following decompressive lumbar laminectomy for degenerative spinal stenosis: a comparison of patients undergoing concomitant arthrodesis versus decompression alone. J Neurosurg 1996;**85**:793-802.
14. Johnsson KE, Willner S, Johnsson K. Postoperative instability after decompression for lumbar spinal stenosis. Spine 1986;**11**:107-10.
15. Tuite GF, Doran SE, Stern JD, et al. Outcome after laminectomy for lumbar spinal stenosis. Part II: Radiographic changes and clinical correlations. J Neurosurg 1994;**81**:707-15.
16. Thome C, Zevgaridis D, Leheta O, et al. Outcome after less-invasive decompression of lumbar spinal stenosis: a randomized comparison of unilateral laminotomy, bilateral laminotomy, and laminectomy. J Neurosurg Spine 2005;**3**(2):129-41.
17. Mayer HM. *Minimally Invasive Spine Surgery. A Surgical Manual*: Springer, 2005.
18. Oppenheimer JH, DeCastro I, McDonnell DE. Minimally invasive spine technology and minimally invasive spine surgery: a historical review. Neurosurg Focus 2009;**27**(3):E9.
19. Nerland US, Jakola AS, Solheim O, et al. Minimally invasive decompression versus open laminectomy for central stenosis of the lumbar spine: pragmatic comparative effectiveness study. (1756-1833 (Electronic)).
20. Hu SS, Tribus CB, Diab M, et al. Spondylolisthesis and spondylolysis. J Bone Joint Surg Am 2008;**90**(3):656-71.
21. Meyerding HW. Spondylolisthesis. Surg Gynecol Obstet 1932;**54**:371-77.

For peer review only - http://bmjopen.bmj.com/site/about/guidelines.xhtml

22. Aryanpur J, Ducker T. Multilevel lumbar laminotomies: an alternative to laminectomy in the treatment of lumbar stenosis. Neurosurgery 1990;**26**(3):429-32; discussion 33.
23. Hutchinson PJ, Laing RJ, Waran V, et al. Assessing outcome in lumbar disc surgery using patient completed measures. Br J Neurosurg 2000;**14**(3):195-9.
24. Roland M, Morris R. A study of the natural history of low-back pain. Part II: development of guidelines for trials of treatment in primary care. Spine (Phila Pa 1976) 1983;**8**(2):145-50.
25. Roland M, Morris R. A study of the natural history of back pain. Part I: development of a reliable and sensitive measure of disability in low-back pain. Spine (Phila Pa 1976) 1983;**8**(2):141-4.
26. Brouwer S, Kuijer W, Dijkstra PU, et al. Reliability and stability of the Roland Morris Disability Questionnaire: intra class correlation and limits of agreement. Disabil Rehabil 2004;**26**(3):162-5.
27. Smeets R, Koke A, Lin CW, et al. Measures of function in low back pain/disorders: Low Back Pain Rating Scale (LBPRS), Oswestry Disability Index (ODI), Progressive Isoinertial Lifting Evaluation (PILE), Quebec Back Pain Disability Scale (QBPDS), and Roland-Morris Disability Questionnaire (RDQ). Arthritis Care Res (Hoboken) 2011;**63 Suppl 11**:S158-73.
28. Fairbank JC, Pynsent PB. The Oswestry Disability Index. Spine (Phila Pa 1976) 2000;**25**(22):2940-52; discussion 52.
29. van Hooff ML, Spruit M, Fairbank JC, et al. The Oswestry Disability Index (version 2.1a): validation of a Dutch language version. Spine (Phila Pa 1976) 2015;**40**(2):E83-90.
30. Williamson A, Hoggart B. Pain: a review of three commonly used pain rating scales. J Clin Nurs 2005;**14**(7):798-804.
31. Chapman JR, Norvell DC, Hermsmeyer JT, et al. Evaluating common outcomes for measuring treatment success for chronic low back pain. Spine (Phila Pa 1976) 2011;**36**(21 Suppl):S54-68.
32. Bombardier C. Outcome assessments in the evaluation of treatment of spinal disorders: summary and general recommendations. Spine (Phila Pa 1976) 2000;**25**(24):3100-3.
33. Gautschi OP, Corniola MV, Joswig H, et al. The timed up and go test for lumbar degenerative disc disease. J Clin Neurosci 2015;**22**(12):1943-8.
34. Gautschi OP, Smoll NR, Corniola MV, et al. Validity and Reliability of a Measurement of Objective Functional Impairment in Lumbar Degenerative Disc Disease: The Timed Up and Go (TUG) Test. Neurosurgery 2016;**79**(2):270-8.
35. Gautschi OP, Joswig H, Corniola MV, et al. Pre- and postoperative correlation of patient-reported outcome measures with standardized Timed Up and Go (TUG) test results in lumbar degenerative disc disease. Acta Neurochir (Wien) 2016;**158**(10):1875-81.
36. Victor ES, Marc LS. The five-repetition sit-to-stand test: evaluation of a simple and objective tool for the assessment of degenerative pathologies of the lumbar spine. 2018;**29**(4):380.
37. Jones SE, Kon SS, Canavan JL, et al. The five-repetition sit-to-stand test as a functional outcome measure in COPD. Thorax 2013;**68**(11):1015-20.
38. Grelat M, Gouteron A, Casillas JM, et al. Walking Speed as an Alternative Measure of Functional Status in Patients with Lumbar Spinal Stenosis. (1878-8769 (Electronic)).
39. Stienen MN, Gautschi OP, Staartjes VE, et al. Reliability of the 6-minute walking test smartphone application. (1547-5646 (Electronic)).
40. Tosic L, Goldberger E, Maldaner N, et al. Normative data of a smartphone app-based 6-minute walking test, test-retest reliability, and content validity with patient-reported outcome measures. (1547-5646 (Electronic)).
41. Niggemann P, Kuchta J, Grosskurth D, et al. Spondylolysis and isthmic spondylolisthesis: impact of vertebral hypoplasia on the use of the Meyerding classification. The British journal of radiology 2012;**85**(1012):358-62.
42. Herdman M, Gudex C, Lloyd A, et al. Development and preliminary testing of the new five-level version of EQ-5D (EQ-5D-5L). Qual Life Res 2011;**20**(10):1727-36.

For peer review only - http://bmjopen.bmj.com/site/about/guidelines.xhtml

43. M MV, K MV, S MAAE, et al. Dutch Tariff for the Five-Level Version of EQ-5D. Value Health 2016;**19**(4):343-52.
44. Chan AW, Tetzlaff JM, Altman DG, et al. SPIRIT 2013 statement: defining standard protocol items for clinical trials. Ann Intern Med 2013;**158**(3):200-7.
45. Jordan K, Dunn KM, Lewis M, et al. A minimal clinically important difference was derived for the Roland-Morris Disability Questionnaire for low back pain. J Clin Epidemiol 2006;**59**(1):45-52.
46. Versteegh M, Knies S, Brouwer W. From Good to Better: New Dutch Guidelines for Economic Evaluations in Healthcare. Pharmacoeconomics 2016;**34**(11):1071-74.
47. White IR, Royston P, Wood AM. Multiple imputation using chained equations: Issues and guidance for practice. Stat Med 2011;**30**(4):377-99.
48. Overdevest GM, Moojen WA, Arts MP, et al. Management of lumbar spinal stenosis: a survey among Dutch spine surgeons. Acta Neurochir (Wien) 2014;**156**(11):2139-45.
49. Overdevest GM, Jacobs W Fau - Vleggeert-Lankamp C, Vleggeert-Lankamp C Fau - Thomé C, et al. Effectiveness of posterior decompression techniques compared with conventional laminectomy for lumbar stenosis. (1469-493X (Electronic)).
50. Hermansen EA-O, Austevoll IM, Romild UK, et al. Study-protocol for a randomized controlled trial comparing clinical and radiological results after three different posterior decompression techniques for lumbar spinal stenosis: the Spinal Stenosis Trial (SST) (part of the NORDSTEN Study). (1471-2474 (Electronic)).
51. Soliman MAR, Ali A. Decompression of lumbar canal stenosis with a bilateral interlaminar versus classic laminectomy technique: a prospective randomized study. Neurosurg Focus 2019;**46**(5):E3.

AUTHOR'S CONTRIBUTION

J.R.J. Arjun Sharma: JAS

P.S. Gadjradj: PG

Wouter Mooijen: WM

Wilco Peul: WP

Maurits van Tulder: MvT

B.S. Harhangi: BH

JAS, PG contributed to drafting article and took the lead in writing the manuscript. All authors provided critical feedback and helped shape the research and manuscript.

JAS, PG, WM, WP and BH devised the main conceptual ideas and designed the study.

MvT designed the statistical analysis and the economic evaluation.

PG and BH provided funding for conducting this study. JAS, PG, WM, WP, MvT and BH contributed to the study protocol.

For peer review only - http://bmjopen.bmj.com/site/about/guidelines.xhtml

APPENDIX 1

Informed consent materials/form

Interarcuaire decompressie versus laminectomie bij neurogene claudicatie.

- Ik heb de informatiebrief gelezen. Ook kon ik vragen stellen. Mijn vragen zijn voldoende beantwoord. Ik had genoeg tijd om te beslissen of ik meedoe.
- Ik weet dat meedoen vrijwillig is. Ook weet ik dat ik op ieder moment kan beslissen om toch niet mee te doen of te stoppen met het onderzoek. Daarvoor hoef ik geen reden te geven.
- Ik geef toestemming voor het informeren van mijn huisarts/de specialist die mij behandelt dat ik meedoe aan dit onderzoek.
- Ik geef toestemming voor het opvragen van informatie bij de specialist die mij behandelt over de wervelkanaalstenose.
- Ik geef toestemming voor het verzamelen en gebruiken van mijn gegevens voor de beantwoording van de onderzoeksvraag in dit onderzoek.
- Ik weet dat voor de controle van het onderzoek sommige mensen toegang tot al mijn gegevens kunnen krijgen. Die mensen staan vermeld in deze informatiebrief. Ik geef toestemming voor die inzage door deze personen.

<voor zover van toepassing:>

- Ik geef toestemming voor het informeren van mijn huisarts en/of behandelend specialist van onverwachte bevindingen die van belang (kunnen) zijn voor mijn gezondheid.
- Ik geef □ **wel**

  □ **geen**

  toestemming om mijn persoonsgegevens langer te bewaren en te gebruiken voor toekomstig onderzoek op het gebied van mijn aandoening en de onderzochte behandelwijze
- Ik geef □ **wel**

  □ **geen**

  toestemming om mij na dit onderzoek opnieuw te benaderen voor een vervolgonderzoek.
- Ik wil □ **wel**

  □ **niet**

  geïnformeerd worden over welke behandeling ik heb gehad/in welke groep ik zat.

- Ik wil meedoen aan dit onderzoek.

For peer review only

Naam proefpersoon:

Handtekening: Datum : __ / __ / __

Ik verklaar dat ik deze proefpersoon volledig heb geïnformeerd over het genoemde onderzoek.

Als er tijdens het onderzoek informatie bekend wordt die de toestemming van de proefpersoon zou kunnen beïnvloeden, dan breng ik hem/haar daarvan tijdig op de hoogte.

Naam onderzoeker (of diens vertegenwoordiger):

Handtekening: Datum: __ / __ / __

Aanvullende informatie is gegeven door:

Naam:

Functie:

Handtekening: Datum: __ / __ / __

----

* Doorhalen wat niet van toepassing is.

For peer review only

Reporting checklist for protocol of a clinical trial.

Based on the SPIRIT guidelines.

Instructions to authors

Complete this checklist by entering the page numbers from your manuscript where readers will find each of the items listed below.

Your article may not currently address all the items on the checklist. Please modify your text to include the missing information. If you are certain that an item does not apply, please write "n/a" and provide a short explanation.

Upload your completed checklist as an extra file when you submit to a journal.

In your methods section, say that you used the SPIRITreporting guidelines, and cite them as:

Chan A-W, Tetzlaff JM, Altman DG, Laupacis A, Gøtzsche PC, Krleža-Jerić K, Hróbjartsson A, Mann H, Dickersin K, Berlin J, Doré C, Parulekar W, Summerskill W, Groves T, Schulz K, Sox H, Rockhold FW, Rennie D, Moher D. SPIRIT 2013 Statement: Defining standard protocol items for clinical trials. Ann Intern Med. 2013;158(3):200-207

		Reporting Item	Page Number
**Administrative information**			
Title	#1	Descriptive title identifying the study design, population, interventions, and, if applicable, trial acronym	1

For peer review only

Trial registration	#2a	Trial identifier and registry name. If not yet registered, name of intended registry	1
Trial registration: data set	#2b	All items from the World Health Organization Trial Registration Data Set	1
Protocol version	#3	Date and version identifier	1
Funding	#4	Sources and types of financial, material, and other support	1
Roles and responsibilities: contributorship	#5a	Names, affiliations, and roles of protocol contributors	1 and 24
Roles and responsibilities: sponsor contact information	#5b	Name and contact information for the trial sponsor	1
Roles and responsibilities: sponsor and funder	#5c	Role of study sponsor and funders, if any, in study design; collection, management, analysis, and interpretation of data; writing of the report; and the decision to submit the report for publication, including whether they will have ultimate authority over any of these activities	1
Roles and responsibilities: committees	#5d	Composition, roles, and responsibilities of the coordinating centre, steering committee, endpoint adjudication committee, data management team, and	n/a

For peer review only

		other individuals or groups overseeing the trial, if applicable (see Item 21a for data monitoring committee)	
**Introduction**			
Background and rationale	#6a	Description of research question and justification for undertaking the trial, including summary of relevant studies (published and unpublished) examining benefits and harms for each intervention	5
Background and rationale: choice of comparators	#6b	Explanation for choice of comparators	5-6
Objectives	#7	Specific objectives or hypotheses	6
Trial design	#8	Description of trial design including type of trial (eg, parallel group, crossover, factorial, single group), allocation ratio, and framework (eg, superiority, equivalence, non-inferiority, exploratory)	6
**Methods: Participants, interventions, and outcomes**			
Study setting	#9	Description of study settings (eg, community clinic, academic hospital) and list of countries where data will be collected. Reference to where list of study sites can be obtained	7

For peer review only

Eligibility criteria	#10	Inclusion and exclusion criteria for participants. If applicable, eligibility criteria for study centres and individuals who will perform the interventions (eg, surgeons, psychotherapists)	7-8
Interventions: description	#11a	Interventions for each group with sufficient detail to allow replication, including how and when they will be administered	8-9
Interventions: modifications	#11b	Criteria for discontinuing or modifying allocated interventions for a given trial participant (eg, drug dose change in response to harms, participant request, or improving / worsening disease)	n/a
Interventions: adherance	#11c	Strategies to improve adherence to intervention protocols, and any procedures for monitoring adherence (eg, drug tablet return; laboratory tests)	n/a
Interventions: concomitant care	#11d	Relevant concomitant care and interventions that are permitted or prohibited during the trial	9
Outcomes	#12	Primary, secondary, and other outcomes, including the specific measurement variable (eg, systolic blood pressure), analysis metric (eg, change from baseline, final value, time to event), method of aggregation (eg, median, proportion), and time point for each outcome. Explanation of the clinical relevance of chosen efficacy and harm outcomes is strongly recommended	9-14

For peer review only

Participant timeline	#13	Time schedule of enrolment, interventions (including any run-ins and washouts), assessments, and visits for participants. A schematic diagram is highly recommended (see Figure)	15
Sample size	#14	Estimated number of participants needed to achieve study objectives and how it was determined, including clinical and statistical assumptions supporting any sample size calculations	16
Recruitment	#15	Strategies for achieving adequate participant enrolment to reach target sample size	n/a
**Methods: Assignment of interventions (for controlled trials)**			
Allocation: sequence generation	#16a	Method of generating the allocation sequence (eg, computer-generated random numbers), and list of any factors for stratification. To reduce predictability of a random sequence, details of any planned restriction (eg, blocking) should be provided in a separate document that is unavailable to those who enrol participants or assign interventions	16
Allocation concealment mechanism	#16b	Mechanism of implementing the allocation sequence (eg, central telephone; sequentially numbered, opaque,	16

For peer review only

		sealed envelopes), describing any steps to conceal the sequence until interventions are assigned	
Allocation: implementation	#16c	Who will generate the allocation sequence, who will enrol participants, and who will assign participants to interventions	16
Blinding (masking)	#17a	Who will be blinded after assignment to interventions (eg, trial participants, care providers, outcome assessors, data analysts), and how	16
Blinding (masking): emergency unblinding	#17b	If blinded, circumstances under which unblinding is permissible, and procedure for revealing a participant's allocated intervention during the trial	16
**Methods: Data collection, management, and analysis**			
Data collection plan	#18a	Plans for assessment and collection of outcome, baseline, and other trial data, including any related processes to promote data quality (eg, duplicate measurements, training of assessors) and a description of study instruments (eg, questionnaires, laboratory tests) along with their reliability and validity, if known. Reference to where data collection forms can be found, if not in the protocol	16-17

For peer review only

Data collection plan: retention	#18b	Plans to promote participant retention and complete follow-up, including list of any outcome data to be collected for participants who discontinue or deviate from intervention protocols	17
Data management	#19	Plans for data entry, coding, security, and storage, including any related processes to promote data quality (eg, double data entry; range checks for data values). Reference to where details of data management procedures can be found, if not in the protocol	17
Statistics: outcomes	#20a	Statistical methods for analysing primary and secondary outcomes. Reference to where other details of the statistical analysis plan can be found, if not in the protocol	17-19
Statistics: additional analyses	#20b	Methods for any additional analyses (eg, subgroup and adjusted analyses)	17-19
Statistics: analysis population and missing data	#20c	Definition of analysis population relating to protocol non-adherence (eg, as randomised analysis), and any statistical methods to handle missing data (eg, multiple imputation)	18

Methods: Monitoring

Data monitoring: formal committee	#21a	Composition of data monitoring committee (DMC); summary of its role and reporting structure; statement of whether it is independent from the sponsor and competing interests; and reference to where further	19

For peer review only

		details about its charter can be found, if not in the protocol. Alternatively, an explanation of why a DMC is not needed	
Data monitoring: interim analysis	#21b	Description of any interim analyses and stopping guidelines, including who will have access to these interim results and make the final decision to terminate the trial	19
Harms	#22	Plans for collecting, assessing, reporting, and managing solicited and spontaneously reported adverse events and other unintended effects of trial interventions or trial conduct	20
Auditing	#23	Frequency and procedures for auditing trial conduct, if any, and whether the process will be independent from investigators and the sponsor	n/a
**Ethics and dissemination**			
Research ethics approval	#24	Plans for seeking research ethics committee / institutional review board (REC / IRB) approval	19
Protocol amendments	#25	Plans for communicating important protocol modifications (eg, changes to eligibility criteria, outcomes, analyses) to relevant parties (eg, investigators, REC / IRBs, trial participants, trial registries, journals, regulators)	19

For peer review only

Consent or assent	#26a	Who will obtain informed consent or assent from potential trial participants or authorised surrogates, and how (see Item 32)	20
Consent or assent: ancillary studies	#26b	Additional consent provisions for collection and use of participant data and biological specimens in ancillary studies, if applicable	n/a
Confidentiality	#27	How personal information about potential and enrolled participants will be collected, shared, and maintained in order to protect confidentiality before, during, and after the trial	20
Declaration of interests	#28	Financial and other competing interests for principal investigators for the overall trial and each study site	1 (n/a)
Data access	#29	Statement of who will have access to the final trial dataset, and disclosure of contractual agreements that limit such access for investigators	20
Ancillary and post trial care	#30	Provisions, if any, for ancillary and post-trial care, and for compensation to those who suffer harm from trial participation	20
Dissemination policy: trial results	#31a	Plans for investigators and sponsor to communicate trial results to participants, healthcare professionals, the public, and other relevant groups (eg, via publication, reporting in results databases, or other data sharing arrangements), including any publication restrictions	20

For peer review only

Dissemination policy: authorship	#31b	Authorship eligibility guidelines and any intended use of professional writers	n/a
Dissemination policy: reproducible research	#31c	Plans, if any, for granting public access to the full protocol, participant-level dataset, and statistical code	n/a

Appendices

Informed consent materials	#32	Model consent form and other related documentation given to participants and authorised surrogates	25-26
Biological specimens	#33	Plans for collection, laboratory evaluation, and storage of biological specimens for genetic or molecular analysis in the current trial and for future use in ancillary studies, if applicable	n/a

The SPIRIT checklist is distributed under the terms of the Creative Commons Attribution License CC-BY-ND 3.0. This checklist was completed on 05. January 2020 using https://www.goodreports.org/, a tool made by the EQUATOR Network in collaboration with Penelope.ai

For peer review only
